# Reorganization of the Endosomal System in *Salmonella*-Infected Cells: The Ultrastructure of *Salmonella*-Induced Tubular Compartments

**DOI:** 10.1371/journal.ppat.1004374

**Published:** 2014-09-25

**Authors:** Viktoria Krieger, David Liebl, Yuying Zhang, Roopa Rajashekar, Petr Chlanda, Katrin Giesker, Deepak Chikkaballi, Michael Hensel

**Affiliations:** 1 Abteilung Mikrobiologie, Universität Osnabrück, Osnabrück, Germany; 2 Cell Biology and Biophysics Unit, European Molecular Biology Laboratory Heidelberg, Heidelberg, Germany; 3 Mikrobiologisches Institut, Universitätsklinikum Erlangen, Erlangen, Germany; Stanford University School of Medicine, United States of America

## Abstract

During the intracellular life of *Salmonella enterica*, a unique membrane-bound compartment termed *Salmonella*-containing vacuole, or SCV, is formed. By means of translocated effector proteins, intracellular *Salmonella* also induce the formation of extensive, highly dynamic membrane tubules termed *Salmonella*-induced filaments or SIF. Here we report the first detailed ultrastructural analyses of the SCV and SIF by electron microscopy (EM), EM tomography and live cell correlative light and electron microscopy (CLEM). We found that a subset of SIF is composed of double membranes that enclose portions of host cell cytosol and cytoskeletal filaments within its inner lumen. Despite some morphological similarities, we found that the formation of SIF double membranes is independent from autophagy and requires the function of the effector proteins SseF and SseG. The lumen of SIF network is accessible to various types of endocytosed material and our CLEM analysis of double membrane SIF demonstrated that fluid phase markers accumulate only between the inner and outer membrane of these structures, a space continual with endosomal lumen. Our work reveals how manipulation of the endosomal membrane system by an intracellular pathogen results in a unique tubular membrane compartmentalization of the host cell, generating a shielded niche permissive for intracellular proliferation of *Salmonella*.

## Author Summary

*Salmonella enterica* is an invasive, facultative intracellular bacterial pathogen. Within mammalian host cells, *Salmonella* inhabits a specialized membrane-bound compartment, the *Salmonella*-containing vacuole (SCV), redirects host cell vesicular transport and massively remodels the endosomal system. These activities depend on the function of a type III secretion system and its translocated effector proteins. Intracellular *Salmonella* induces several types of tubular compartments termed *Salmonella*-induced tubules (SIT), but the biogenesis and biological function of SIT is only partially understood. Our work combines live cell imaging with correlative light and electron microscopy to provide ultrastructural insight into SIT. We report that SIT emerge as single membrane tubules that convert into double membrane tubules entrapping cytosol and cytoskeletal filaments. Labeling of the endosomal compartment and cytochemistry demonstrate that the space between inner and outer SIT membrane is composed of internalized material and connected to *Salmonella* within the SCV. The effector proteins SseF and SseG translocated by intracellular *Salmonella* are essential for the conversion of single to double membrane SIT. These findings challenge current models for the intracellular lifestyle of *Salmonella* and the composition of its intracellular habitat.

## Introduction

Bacterial pathogens have evolved sophisticated mechanisms to modify host cell functions in order to avoid antimicrobial defense or to use host cell-derived material for their own proliferation. Intracellular pathogens evade humoral immune responses of the host by hiding inside cells of the host organism, using these cells to support their own proliferation and to disseminate within the host organism [1]. These activities require the manipulation of the normal host cell processes to avoid killing by the host cell and to establish a replication-permissive intracellular niche.

One group of intracellular bacteria resides in specialized membrane compartments that derive from the endosomal system of the host cell. *Salmonella enterica* is a facultative intracellular pathogen that modifies eukaryotic host cells in order to establish a unique parasitophorous vacuole, the *Salmonella*-containing vacuole or SCV [2], [3]. The SCV is a membrane-bound compartment that allows the survival and replication of *S. enterica* in a variety of mammalian host cells. At later time points after infection, the SCV acquires certain characteristics of late endosomal/lysosomal compartments such as (i) the presence of a subset of Rab GTPases, such as Rab7, (ii) lysosomal glycoproteins (lgp), such as LAMP1, (iii) acidification, and (iv) juxtanuclear positioning. However, the SCV remains permissive for intracellular replication of *Salmonella*. Several studies investigated the interaction of the SCV with the host cell endosomal system and indicate that various types of interaction take place during the maturation of this specialized compartment [4]–[7]. One unique phenotype resulting from these interactions is the induction of long tubular membrane compartments extending from the SCV, termed *Salmonella*-induced filaments or SIF [8].

SIF were first observed in *Salmonella*-infected epithelial cells, but we extended this observation also to phagocytic cells [9]. SIF are characterized by presence of specific protein markers similar to those on the SCV membrane and exhibit dynamic properties. The formation of SIF in epithelial cells starts from 3–4 h after invasion by fusion events of the SCV with endosomal vesicles. These early SIF are highly dynamic tubules which often exhibit a thinner appearing part at the distal end, termed leading SIF (LS), in comparison to the proximal part, termed trailing SIF (TS). From 8 h post infection (p.i.) a complex tubular network is established which appears stable and less dynamic. [9], [10].

Despite a large body of work on the intracellular activities of *Salmonella*, the biogenesis and function of SIF are still enigmatic. In addition, recently distinct tubular membrane compartments have been identified induced by intracellular *Salmonella*: Golgi-derived *Salmonella*-induced SCAMP3 tubules (SIST) [11] and LAMP1-negative tubules (LNT) in cells infected with a *sifA sopD2* double mutant strain [12]. Throughout this paper we will use the general term *Salmonella*-induced tubules (SIT) which does not discriminate between SIF, SIST, LNT or another (un)classified subtype of SIF. Characteristic features of SIT are summarized in Table 1.

**Table 1:** Features of *Salmonella*-induced tubules (SIT).

Type	Marker proteins	Time of formation p.i. (maximum)	Reference
**SIF** (*Salmonella*-induced filaments)	lgp, Rab7, vATPase, LBPA, cholesterol	3–16 h (8–16 h)	[4]–[12]
	SPI2-T3SS effectors		
**SIST** (*Salmonella*-induced SCAMP3 tubules)	SCAMP3	8–14 h (14 h)	[11]
	SPI2-T3SS effectors		
**LNT** (LAMP1-negative tubules)	vATPase, cholesterol	3–16 (16 h)	[12]
	SPI2-T3SS effectors		

Of central importance for the intracellular lifestyle of *Salmonella* is the function of the type III secretion system (T3SS) encoded by *Salmonella* Pathogenicity Island 2 (SPI2). Intracellular *Salmonella* deploy the SPI2-T3SS to translocate a set of effector proteins across the membrane of SCV [2]. Collectively, these effector proteins enable the intracellular survival and proliferation of *Salmonella*. For the majority of SPI2-T3SS effectors the exact mode of action and molecular targets in the host cell remain unknown. However, some effectors are likely relevant for both, induction of specific intracellular phenotypes and systemic pathogenesis. Formation of SIF requires at least five *Salmonella* effector proteins, i.e. SifA, SseF, SseG, SopD2 and PipB2, and the integrity of the microtubule cytoskeleton [13], [14]. The most severe phenotype is mediated by the SPI2 effector SifA [15]. Mutant strains lacking SifA are highly attenuated in intracellular replication and systemic virulence. Bacteria deficient in *sifA* fail to induce SIF and escape into the cytoplasm of the host cell due to a loss of SCV membrane integrity [15]. SseF and SseG also contribute to the intracellular lifestyle, although the defects in intracellular replication of the corresponding mutant strains are less pronounced compared to *sifA* or SPI2-T3SS deficient strains.

The strict correlation between intracellular fitness of *Salmonella* and its ability to form SIF prompted us to investigate the nature of these tubular endosomal compartments in *Salmonella*-infected cells. We examined the biogenesis of the SCV and *Salmonella*-induced tubular compartments by a combination of confocal laser scanning microscopy (CLSM) of live infected cells, followed by ultrastructural analyses by transmission electron microscopy (TEM) and electron tomography (ET). Our observations based on correlative light and electron microscopy (CLEM) provide a novel view on the intracellular activities of *Salmonella* leading to unique compartmentalization of the host endosomal membranes and we propose new models for SIF biogenesis.

## Results

### Intracellular *Salmonella* induce various types of host cell membrane tubules

We previously reported the ultrastructure of tubular membrane compartments in connection with SCV [9]. In addition to SIF with the characteristic presence of lgp, recent studies revealed additional types of tubular membrane compartments in *Salmonella*-infected cells including SIST [11] and LNT [12]. Here we use the general term ‘*Salmonella*-induced tubules’ or SIT if no further distinction is possible.

**Figure 1:**
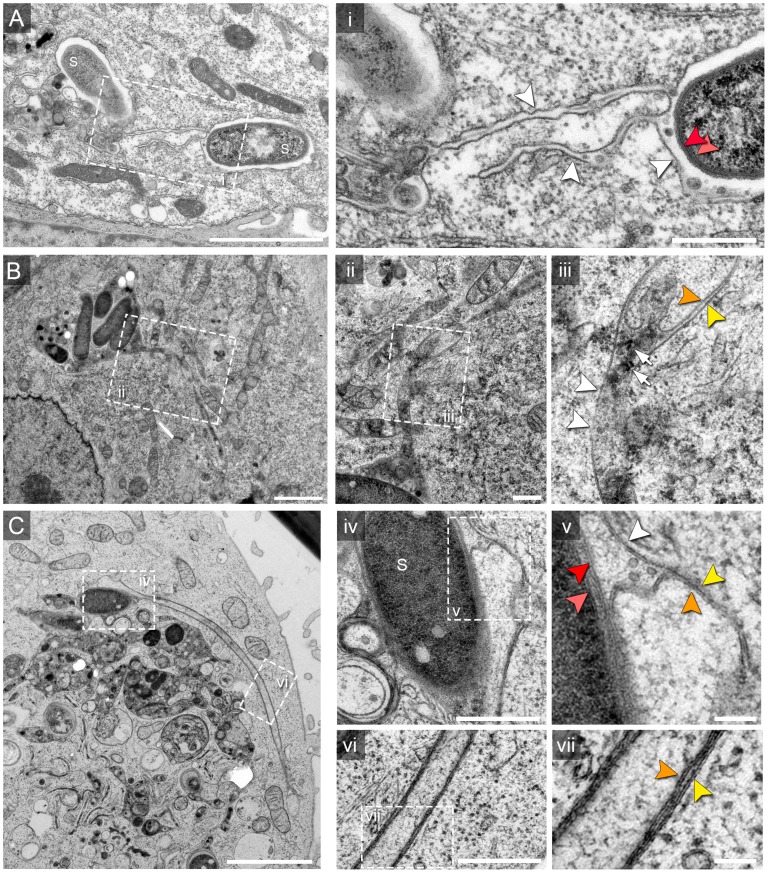
Single and double membrane SIT extend from the SCV in *Salmonella*-infected cells. Electron micrographs of ultrathin sections through HeLa cells infected with *Salmonella enterica* wild type (S). Cells were subjected to HPF-FS at 10 h post infection (p.i.). Representative micrographs of SCV and SIT are shown. A) Example of thin single-membrane SIT interconnecting two SCV B) Cells were pre-loaded with BSA-coupled 10 nm colloidal gold particles to track lumen of endosomes and luminal space of SCV/SIT. Micrograph shows an example of SCV connected to a single-membrane SIT. The SIT lumen is filled with electron-dense content and BSA-gold particles (black dots indicated by white arrows) (iii). Note that parts of SIT appear as double membrane compartments. C) Double membrane tubular compartment emerging from the SCV. Details of membrane organization of SCV (iv, v) and SIT (vi, vii). Note that central portions of SIT have density identical to the cytosol. White arrowheads indicate single membrane compartments and for double membrane compartments, orange and yellow arrowheads distinguish inner and outer membrane, respectively. Light and dark red arrowheads indicate inner and outer membrane of the *Salmonella* cell envelope, respectively. Scale bars: 2 µm (A, B, C), 500 nm (i, ii, iv, vi), 100 nm (iii, v, vii).

For an overview of possible ultrastructural differences of SIT we first performed conventional TEM of *Salmonella* WT-infected HeLa cells (Figure 1). Ultrathin horizontal sections through flat-embedded cells revealed both, longitudinal and cross-sections through SIT. At the time points of sampling, i.e. 8–12 h p.i., the majority of intracellular WT *Salmonella* (82.7%, N = 208 bacteria) were contained within membrane compartments. These SCV enclosed one or more bacteria into a complex membrane organelle with a lumen enriched in electron-dense granules and multi-lamellar vesicles (Figure 1). Membrane tracing on cross-sections through the SCV revealed a continual ‘outer’ membrane of the SCV which encircled bacteria but also numerous membrane-enclosed bubble-like compartments within the lumen of the SCV (Figure S1). Although these compartments were reminiscent of cytosolic pockets enclosed within the complex 3D structure of the SCV, they may as well result from invaginations of the SCV ‘outer’ membrane.

Extended analyses of ultrathin sections by TEM revealed additionally a variety of SIT morphotypes and an unexpected membrane organization of the tubular compartments (Figure 1). Apart from very thin single membrane SIT of rather uniform diameter of 46±8 nm and moderately electron-dense content (Figure 1Ai), we also observed thicker single membrane SIT that appeared more electron-dense (Figure 1Bii, iii). The latter tubules were characterized by a lumen containing electron-dense granules and vesicles, reminiscent of the luminal content of late endosomes or endolysosomes [16], and will be referred to as type 1 SIT. Most frequently, we observed extended tubules with luminal content of low electron density (Figure 1Cvi, vii). These SIT were delimited by two adjacent membranes which we discriminate as outer and inner membrane. These structures will be referred to as type 2 SIT. Both type 1 and type 2 SIT were also found in the same cells, and in some sections, interconnection between both types of tubules was detected. From observations by light microscopy, *Salmonella* is known to induce tubular networks [8], [11], [12]. We identified and traced the observed tubular structures by TEM throughout serial sections of *Salmonella* WT-infected cells (about 100 sections per cell), indicating also a complex 3D network of SIT. Thus, quantification of the frequency of SIT phenotypes in ultrathin sections by TEM was not feasible given the complexity, size and 3D organization of SIT. However, inspection of TEM ultrathin sections allows an approximation of the frequency of SIT phenotypes. Sections of HeLa cells (N = 124 cells) infected with *Salmonella* WT for 8–12 h revealed that 13.7% of cells displayed type 1 SIT, 74.2% displayed type 2 SIT, and 6.5% of cells showed both SIT types.

Measurements of diameters revealed for type 1 SIT average diameters for single membrane tubules of 120±46 nm (N = 7 cells with 5 measurements per SIT) and for type 2 SIT, the average diameter of double membrane tubules was 221±65 nm (N = 30 cells with 5 measurements per SIT, Figure S2A). The mean distance between inner and outer membrane of the tubule was 31±12 nm. We observed that the diameter of type 2 SIT was rather constant over the segment present in one ultrathin section. Examples of longitudinal sections and cross sections through type 2 SIT are shown in Figure S2.

### Type 2 SIT inner lumen contains cytosolic content and cytoskeletal filaments

**Figure 2:**
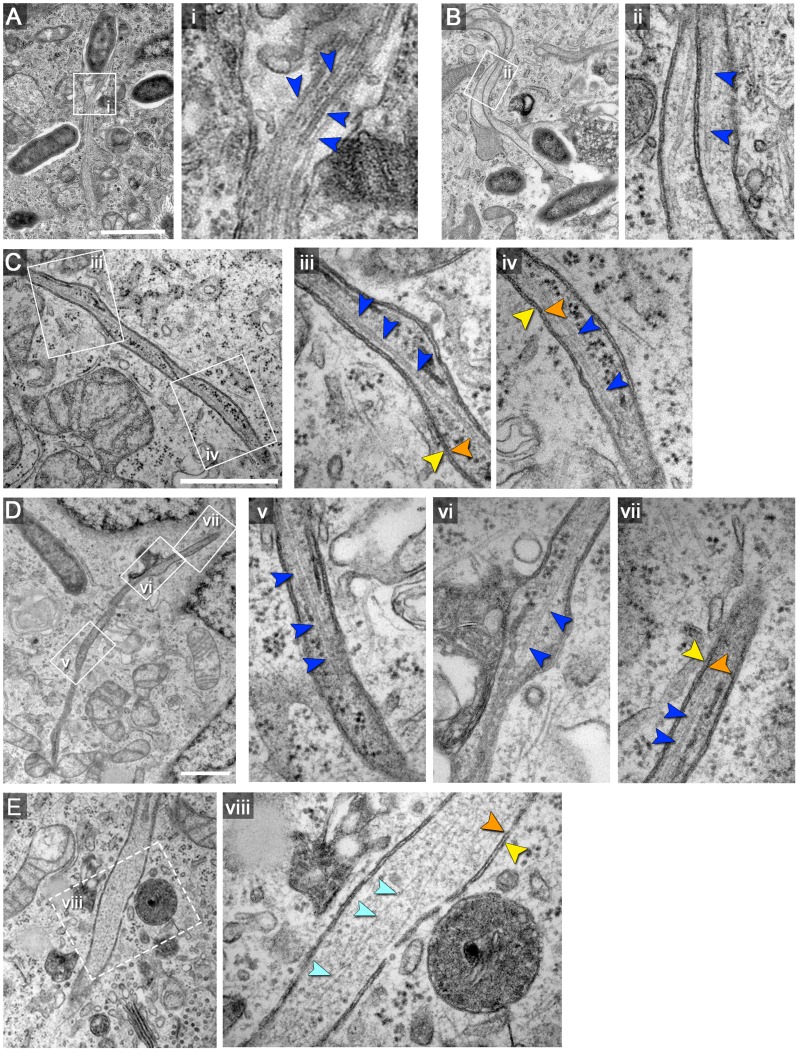
Double membrane SIT contain cytoskeletal filaments and ribosomes. HeLa cells were infected with *S. enterica* WT as for Figure 1. A), B), C) and D) show examples of double-membrane SIT with microtubules, indicated by dark blue arrowheads. Orange and yellow arrowheads indicate inner and outer membranes of SIT, respectively. Measurements of microtubules as shown in i) revealed an average diameter of 22 nm±2.3 nm. Higher magnifications are shown in panel ii to vii. E) shows an example of a double-membrane SIT containing thinner, actin-like filaments (light blue arrowheads). Note that the inner space of all double-membrane SIT (A–E) also contains ribosomes. Scale bars: 500 nm.

The closer examination of type 2 SIT by conventional TEM indicated that the inner lumen of the double membrane tubules contains numerous ribosomes and occasionally also filamentous structures. Filaments of smaller and larger diameter were detected with a distinct morphological similarity to actin filaments and microtubules, respectively (Figure 2). We determined the diameter of the larger filaments as 22.05±2.29 nm (N = 15) which is consistent with the diameter of microtubules (22–24 nm) (Figure 2A–D) [17]. Microtubules were detected only in a subpopulation of sections through type 2 SIT, suggesting that their presence or absence may be related to a certain developmental stage of the tubules. The thinner filaments had diameters of less than 5 nm, but a precise measurement was not possible due to limits of resolution in 40–60 nm sections (Figure 2E). Based on the filament thickness and morphology in accordance with previous observations [17], we propose that the thinner filaments are F-actin microfilaments. Immuno-gold labeling of cytoskeleton in TEM sections was not successful, probably due to insufficient preservation. Thus, our conclusion of F-actin and microtubules within SIT so far is based on the typical ultrastructural morphology. For an unambiguous determination of the composition of the SIF lumen, our future work will make use of genetically encoded tags [18]. Co-localization of SIF and LNT with microtubules [9], [12] and F-actin (DC, KG, MH, unpublished data) was already observed, but light microscopy cannot reveal if cytoskeletal filaments are on the outside of SIT, or within the lumen. Examination of various ultrathin sections of HeLa cells (N = 124 cells) 8–12 h after infection with *Salmonella* WT revealed that 27.2% or 4.3% of type 2 SIT contain microtubules or F-actin filaments, respectively. The density of cytoskeletal filaments inside type 2 SIT was variable, but some compartments appeared filled with a large amount of filaments (Figure 2Ai). The ultrastructure-based localization of cytoskeletal filaments both outside [9] and inside type 2 SIT (this paper) clearly emphasizes their prominent role in the biogenesis of SIT.

These findings suggest that the interior space of type 2 SIT is either continuous with the host cell cytosol or represents a partial volume of cytosol enwrapped by double membranes during formation of the tubule.

### SIF lumen is accessible to various types of endosomal content

**Figure 3:**
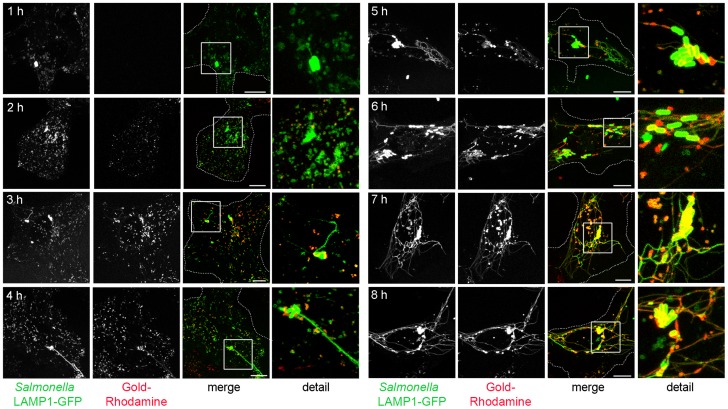
The SIF network is accessible to various types of endosomal cargo. HeLa cells expressing LAMP1-GFP (green) were infected with *Salmonella* expressing GFP (green, rod-shaped structures). Pulse-chase experiments were performed by addition of 10 nm gold Nanoparticles conjugated with BSA-Rhodamine (red) at various time points prior and after infection. Live cell imaging of infected cells was performed and representative cells are shown. LAMP1-GFP-positive tubules emerged at 3 to 4 h p.i. Note the almost complete co-localization of endosomal cargo with tubular, LAMP1-GFP-positive SIT. Scale bar: 10 µm.

In order to investigate the continuity between the lumen of SIT and endosomes, we probed the intracellular environment of *Salmonella* by pulse-chase experiments in combination with live cell imaging. Our previous analyses demonstrated that BSA-Rhodamine-conjugated gold nanoparticles are a useful experimental tool that efficiently accumulate and label endosomal compartments [19]. This tracer is also suitable for CLEM applications by virtue of electron-dense gold particles of defined size, as well as the possibility to use Rhodamine for photo-conversion of diaminobenzidine (DAB) [20]. Additionally, in order to obtain a moderate and consistent expression level of an endosomal membrane marker, we used lentiviral transfection to generate a HeLa cell line constitutively expressing LAMP1-GFP which also localizes to SIF. This cell line was indistinguishable from the parental cell line with respect to the intracellular phenotypes of *Salmonella* (Figure S3, Figure S4, Figure S5) and was therefore used in all microscopic approaches. For fluid tracer experiments, HeLa LAMP1-GFP cells were pulse-chased with BSA-Rhodamine-gold at various times prior or after the infection with *Salmonella* WT and analyzed by live cell imaging (Figure 3). Endocytosed BSA-Rhodamine-gold distributed rapidly within the network of SIF and all LAMP1-positive compartments were also positive for the fluid tracer. Compared to the positive control (co-localization rate of 78.5±1.3% and Pearson's correlation coefficient of 0.82±0.01), LAMP1-GFP and Gold-BSA-Rhodamine showed comparable high co-localization in either non-infected cells (co-localization rate of 80.22±6.7% and Pearson's correlation coefficient of 0.85±0.02) or cells post infection (e.g., at 8 h p.i., co-localization rate of 79.7±3.5% and Pearson's correlation coefficient of 0.74±0.02). We also observed the fluid tracer within the SCV, decorating the bacterial cell body. The intensity of Rhodamine fluorescence increased with the duration of the pulse and consequently with the amount of the marker accumulated in the endosomal system of the cell. In conclusion, these data show that SIF lumen is accessible to endosomal content and support our observation in TEM of the endosomal-like content of type 1 SIT (Figure 1B).

### Correlative light and electron microscopy reveals identity of *Salmonella*-induced filaments

**Figure 4:**
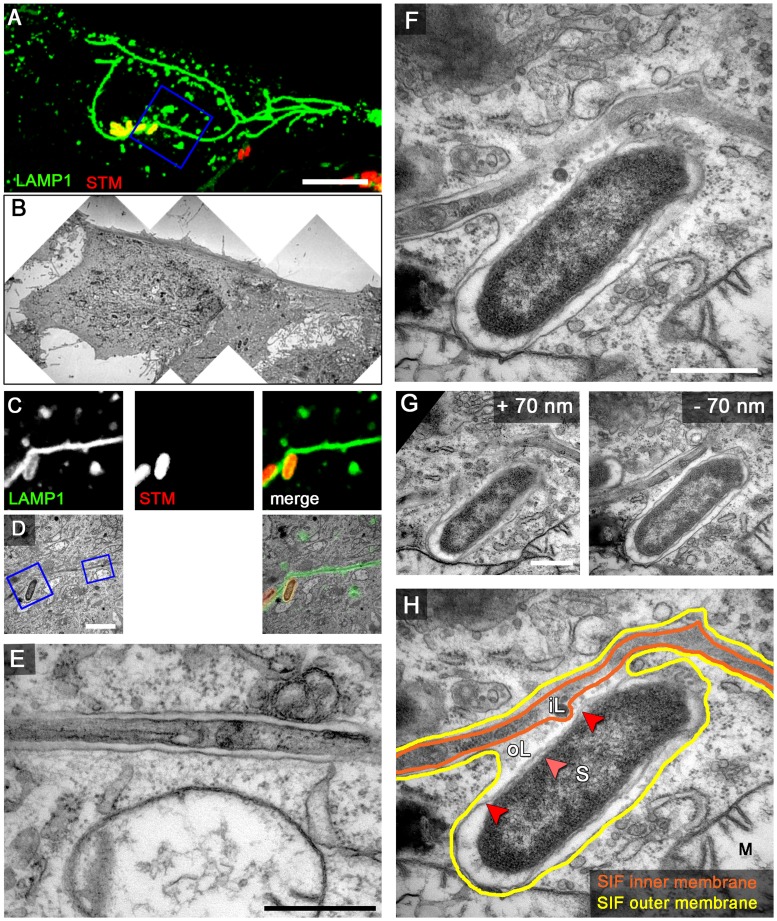
LAMP1-GFP-positive SIF display a double membrane at 8 h p.i. HeLa cells expressing LAMP1-GFP (green) were seeded in Petri dishes with a gridded coverslip and infected with *Salmonella* expressing mCherry (STM, red). Live cell imaging was performed 8 h p.i. to visualize LAMP1-GFP-positive SIF (A, maximum intensity projection [MIP], C, single Z plane). Subsequently, the cells were fixed and processed for CLEM to reveal the ultrastructure of selected cells. Several low magnification images were stitched to visualize the cell morphology (B). Higher magnification images were used to align LM and TEM images (D). Details of a LAMP1-GFP-positive double membrane SIF (E) and an SCV linked to a SIF (F, G) are shown. G) Two additional ultrathin sections show the membrane organization of the SCV and SIF in detail F). H) The inner and outer membrane of a SIF and SCV are outlined in orange and yellow, respectively. Light and dark red arrowheads indicate inner and outer membrane of the *Salmonella* cell envelope, respectively. Labels: S, *Salmonella*; M, mitochondria; iL, inner lumen; oL, outer lumen. A cell representative for 10 biological replicates is shown (1–3 technical replicates with each 2–4 cells). Scale bars: 10 µm (A, B), 2 µm (C, D), 500 nm (E, F, G).

The precise interpretation of the SIF integrity requires the analysis of *Salmonella* infection in living cells [9], [21]. Therefore, we used the HeLa LAMP1-GFP cell line for live cell imaging of infected cells followed by processing for CLEM. This approach allowed us first to follow the integrity and dynamics of LAMP1-GFP-positive tubular structures in living infected cells, and then to investigate the ultrastructural features of the same tubular structures by TEM. We observed that at 8 h after infection with *Salmonella* WT all double membrane type 2 SIT were positive for LAMP1-GFP (Figure 4). Thus, we conclude that type 2 SIT are of late endosomal/lysosomal origin and are identical to the previously described SIF. Any similar tubular compartments were neither observed in non-infected HeLa cells (Figure S6), nor in cells infected with an *ssaV*-deficient strain unable to translocate SPI2-T3SS effector proteins (data not shown).

**Figure 5:**
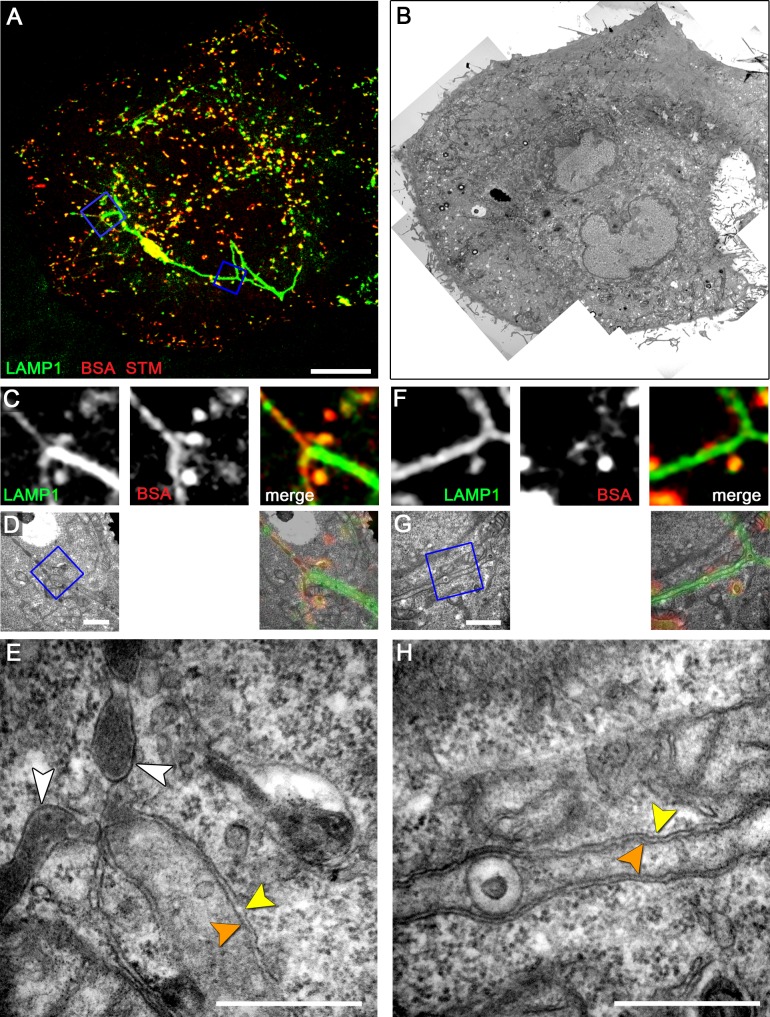
Early-stage SIF in HeLa cells exhibit leading and trailing phenotypes. HeLa expressing LAMP1-GFP (green) were seeded on a Petri dish with gridded coverslip and infected with *Salmonella* WT expressing mCherry (STM, red). After infection, cells were pulse-chased with BSA-Rhodamine (red) 1–3 h p.i. Live cell imaging was performed 4 h p.i. to visualize LAMP1-GFP-positive SIF (A, MIP; C, F, single Z plane). Subsequently, the cells were fixed and processed for CLEM. Several low magnification images were stitched to visualize the cell morphology (B). C–H) CLEM of ROIs showing SIF of various diameter (D, E), and a double membrane SIF with an internal vesicle (G, H). E) A tubular compartment showing the transition from a leading SIF with a single membrane and endosomal content to a trailing SIF with a double membrane and cytosolic content. H) Double membrane SIF with luminal membrane vesicle. Orange and yellow arrowheads indicate inner and outer membranes if double membrane SIF, respectively. The white arrowheads indicate single membrane SIF. A cell representative for three biological replicates is shown (1–3 technical replicates with each 2–4 cells). Scale bars: 10 µm (A, B), 1 µm (C, D, E, G), 500 nm (E, H).

We next analyzed infected cells shortly after onset of SIF formation, i.e. 4–5 h p.i. (Figure 5, Figure S7). Previous work showed that SIF are highly dynamic at this early stage and undergo extension, branching and contraction [9], [10]. Furthermore, in living cells, we frequently observed connections between LAMP1-positive tubules of two kinds: (i) thinner tubules with weak fluorescence intensity often localized at the periphery of the cell and (ii), centrally localized thicker tubules with strong fluorescence intensity connected to SCV (Figure S5). This phenotype was reminiscent of the previously described leading SIF (LS) and trailing SIF (TS) [9], [10]. When observed by light microscopy LS extend further from the distal end of TS. Importantly, frequently detected gradual transitions of LS into TS suggest that they both represent one tubular structure undergoing morphological transition(s). We therefore set out to analyze these morphological features of WT-infected HeLa LAMP1-GFP cells by CLEM, additionally pulse-chased with BSA-Rhodamine. Our results indicated two types of SIF morphologies. SIF representing LS in light microscopy correlated with single membrane SIT of thinner diameter and electron-dense content, similar to type 1 SIT. These were often found in continuum with double membrane SIT of extended diameter and cytoplasmic content, similar to type 2 SIT, correlating with TS in light microscopy (Figure 5). Based on these observations we propose that the dynamic conversion of leading into trailing SIF observed by light microscopy, in fact corresponds to the conversion of a single membrane tubule (type 1 SIT) to a double membrane tubule (type 2 SIT). Consequently, the observed type 1 SIT or LS are of late endosomal/lysosomal origin and serve as precursor of type 2 SIT or TS, while both morphological structures represent LAMP1-positive SIF in *Salmonella* WT-infected cells.

### *Salmonella* induces double membrane SIF in RAW264.7 macrophages

**Figure 6:**
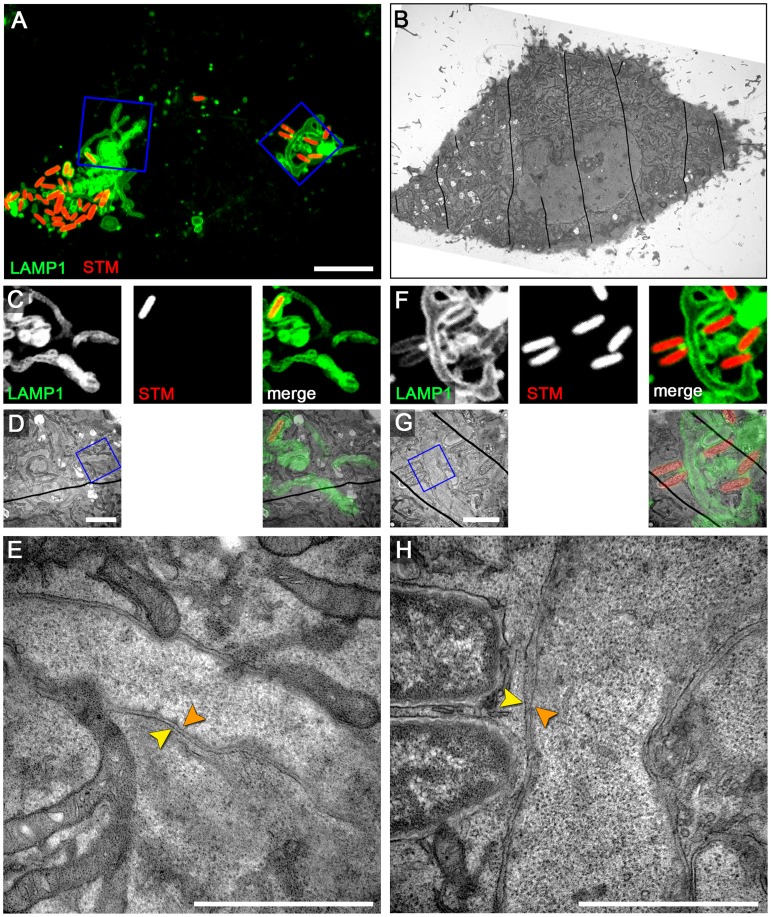
RAW264.7 macrophages infected with *Salmonella* exhibit double membrane SIF. RAW264.7 cells expressing LAMP1-GFP (green) were seeded in Petri dishes with a gridded coverslip and infected with *Salmonella* WT expressing mCherry (STM, red). Live cell imaging was performed 12 h p.i. to visualize LAMP1-GFP-positive SIF (A, MIP; C, F, single Z plane). Subsequently, the cells were fixed and processed for CLEM to reveal the ultrastructure. Several low magnification images were stitched to visualize the cell morphology (B). Higher magnification images were used to align LM and TEM images (D, G). Details of LAMP1-GFP-positive double membrane SIF (E, H) are shown. A cell representative of three biological replicates is shown (1–2 technical replicates with each 2–4 cells). Scale bars: 10 µm (A, B), 2 µm (C, D), 500 nm (E).

To test if the formation of double membrane SIF (type 2 SIT) is restricted to HeLa cells or a more general phenomenon, we analyzed *Salmonella*-infected RAW264.7 macrophages by live cell CLEM. First, a RAW264.7 cell line with stable expression of LAMP1-GFP was generated by lentiviral transfection and the transfection had no influence on the intracellular phenotypes of *Salmonella* (data not shown). To achieve a flat, adherent cell morphology for microscopic studies, the macrophages were stimulated with Interferon-γ (IFNγ). We first investigated non-infected RAW264.7 macrophages activated by IFNγ, since long tubular endosomal compartments were reported for phagocytic cells [22]. CLEM of non-infected activated RAW264.7 cells showed thin LAMP1-GFP-positive single membrane tubules that contain many small vesicles (Figure S8A–E). These tubular structures in RAW264.7 cells were sometimes extending throughout the entire cell. In infected RAW264.7 cells the LAMP1-GFP-positive SIF were identified by virtue of their connections to the SCV. These tubules were substantially thicker and with stronger LAMP1-GFP signal compared to LAMP1-GFP-positive tubules in non-infected cells. Macrophages were infected with stationary phase *Salmonella* to avoid invasion-induced pyroptosis, thus the onset of SIF formation in infected RAW264.7 macrophages is delayed compared to infected HeLa cells [9]. Accordingly, we adjusted time points for microscopic analyses. CLEM of activated and *Salmonella* WT-infected RAW264.7 LAMP1-GFP cells revealed single membrane tubules at 8 h p.i. (Figure S8F–J), comparable to type 1 SIT in HeLa cells at 4 h p.i., as well as double membrane tubules at 12 h p.i. (Figure 6), comparable to type 2 SIT in HeLa cells at 8 h p.i. Pulse-chase experiments with BSA-Rhodamine and subsequent photo-conversion of DAB in infected RAW264.7 macrophages showed similar results as for HeLa cells (data not shown). In summary, these data demonstrate the presence of type 2 SIT in RAW264.7 macrophages comparable to double membrane SIF in HeLa cells, and suggest that induction of double-membrane tubules by intracellular *Salmonella* is a common phenomenon.

### *Salmonella*-induced double membrane tubules are distinct from autophagosomes

Double (and multiple-) membrane compartments are typically observed during the formation of autophagosomes [23], and autophagy has been reported as a host cell factor involved in the control of intracellular *Salmonella* [24]–[27]. We thus tested whether SIT are related to autophagosomes. Microtubule-associated protein 1 light chain 3 (LC3), a mammalian homolog of yeast ATG8, is a well-established marker of autophagy and its proteolytically processed form, LC3-II, associates with membranes of the phagophore, autophagosome and autolysosome [28], [29]. HeLa cells were transfected with GFP-LC3b, infected with *Salmonella* WT and the endosomal system was then labeled with fluorescent fluid-phase marker (Figure S9A). Epifluorescence microscopy of living cells showed that a small fraction of intracellular bacteria (app. 5–10%) was associated with LC3b, a finding in line with previous observations on the cytosolic presence of a subset of intracellular bacteria and the targeting of some of these bacteria by autophagy [30]. Starting at 3–4 h p.i., GFP-LC3b-transfected HeLa cells also showed the presence of SIT, but these were not associated with LC3b at all and the distribution of the GFP-LC3b signal was clearly distinct from the tubular arrangement of the endocytosed fluid tracer (Figure S9A). To further examine the possible involvement of autophagy, we inhibited ATG5, a key regulator of all known human ATG8 homologous [reviewed in 31]. Therefore HeLa LAMP1-GFP cells were transiently transfected with a plasmid encoding mCherry-ATG5-K130R, a dominant-negative form of ATG5 [32], [33]. Transfected cells were infected with *Salmonella* WT, analyzed by light microscopy and prepared for CLEM. Interestingly, CLSM revealed a reduction in SIF formation compared to non-transfected HeLa cells. Non-transfected HeLa cells infected by *Salmonella* WT showed 72.3±3.5% of infected cells with SIF formation. *Salmonella* WT-infected cells previously transfected with dominant-negative ATG5 revealed only 56.6±6.1% SIF formation (Figure S9B). This observation is consistent with work of Birmingham *et al*. [30], who investigated *Salmonella*-infected atg5^−/−^ MEF. However, we observed no obvious changes in SIF morphology. Micrographs also clearly showed that LAMP1-GFP-positive SIF in HeLa cells transfected with mCherry-ATG5-K130R display a double membrane structure similar to type 2 SIT (Figure S9C–F). Based on these observations, we rule out that biogenesis of type 2 SIT is related to formation of autophagosomes and anticipate a distinct mechanism of double membrane formation.

### The outer lumen of double membrane SIF is accessible for fluid phase markers

**Figure 7:**
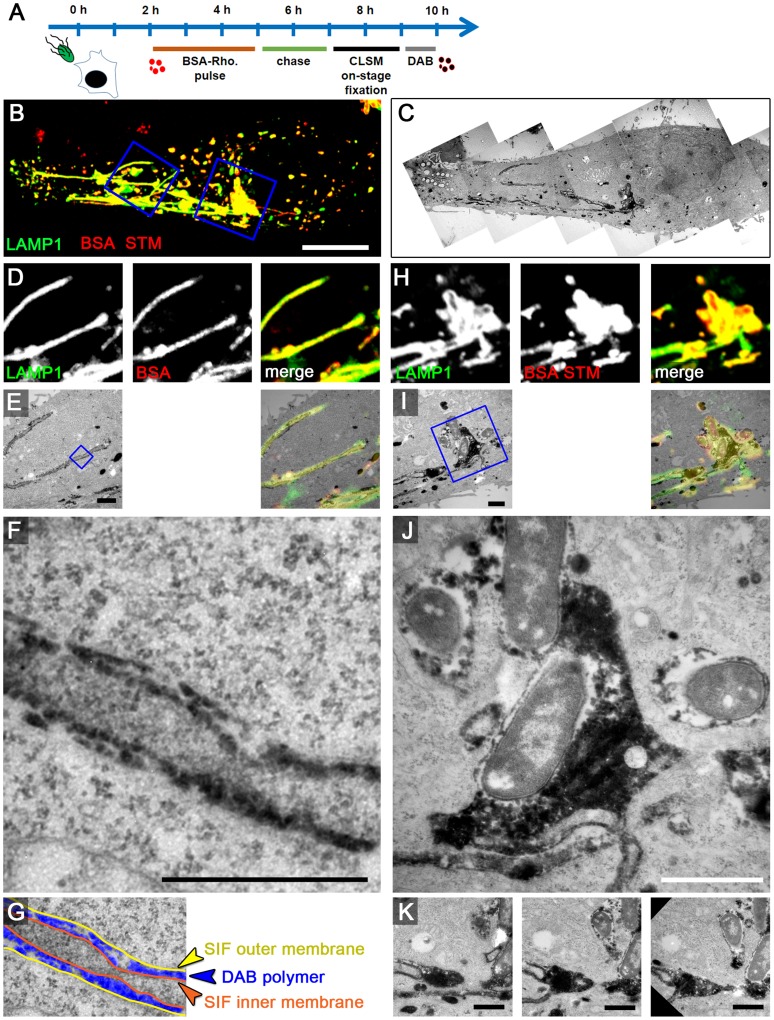
The outer lumen of double membrane SIF is in interchange with endocytosed material. A) Scheme of the experiment. HeLa expressing LAMP1-GFP (green) were seeded on a petri dish with a gridded coverslip. Cells were infected with *Salmonella* WT expressing mCherry (STM, red). BSA-Rhodamine was added as fluid tracer to the medium 2–5 h p.i. After live cell imaging of selected cells at 8 h p.i. by CLSM (B, MIP), cells were immediately fixed on stage. DAB photo-conversion by Rhodamine was performed and cells were prepared for TEM. Several images of the same section were stitched for an overview (C). Details for LAMP1-positive, fluid tracer-labeled SIF (D–G) and SCV with attached SIF (H–K) are shown by correlative live cell CLSM (D, H, single Z plane) and TEM (E–G, I–K) micrographs. F) shows a higher magnification of double membrane SIF, and the pseudocolored micrograph (G) indicates the organization of inner and outer SIF membrane and the DAB polymer deposition in intermembrane lumen. J) shows DAP polymer deposition in direct contact to *Salmonella* within the SCV. Successive sections with several SIF extending from the SCV are shown in K). A cell representative of three biological replicates is shown (1–3 technical replicates with each 2–4 cells). Scale bars: 10 µm (B, C), 1 µm (D, E, H, I, K), 500 nm (F, J).

The observation of double membrane SIF raised the question of the origin of the SIF inner lumen and of the lumen between inner and outer membrane, termed SIF outer lumen. To analyze the lumen of SIF with ultrastructural resolution, we applied pulse-chase experiments with fluid-phase markers suitable for detection by both, CLSM and EM. Although Rhodamine-gold provided intense and uniform labeling of the SIF lumen (Figure 3), the frequency of gold NP in thin sections analyzed by TEM was rather low (<10 particles per SIF in a ultrathin section). As an alternative, we used Rhodamine for the UV light-induced photo-conversion of diaminobenzidine (DAB) [20]. The conversion (oxidation) of DAB leads to a precipitation of an alcohol-insoluble, granular polymer which, after further processing, becomes an electron-dense product detectable by TEM. Alternatively, we used the non-fluorescent horseradish peroxidase (HRP) as a fluid-phase marker catalyzing the conversion of DAB in presence of peroxide [34]. For this analysis, infected cells were first pulse-chased with either BSA-Rhodamine (without gold NP) (Figure 7) or HRP (Figure S10) to allow accumulation of the reagent within endocytic compartments including SCV and SIF, and then subjected to live cell imaging before fixation. The reaction with DAB was performed post fixation prior to sample processing for TEM.

If endocytosed BSA-Rhodamine or HRP interact with SIF, we anticipated that DAB reaction product is located either in the inner lumen, the outer lumen, or both lumen of the double membrane SIT. For LAMP1-GFP-expressing HeLa cells 8 h p.i. and pulse-chase with BSA-Rhodamine, we detected the Rhodamine signal in smaller LAMP1-positive spherical vesicles, as well as inside SIF and within the SCV (Figure 7J), thus showing the same distribution as fluorescent nanoparticles described in Figure 3. However, the correlation with TEM images revealed double membrane tubules with cytosolic content inside the inner lumen (i.e. type 2 SIT) and electron-dense DAB product inside the whole outer lumen. Higher magnification TEM clearly showed that electron-dense DAB product localizes inside the SCV with direct contact to *Salmonella* and inside the outer lumen of double membrane SIF, while the inner lumen was virtually devoid of the marker (Figure 7F).

Comparable results were obtained using HRP. Since no fluorescence signal is present for HRP as a marker, the DAB product was aligned using bright field microscopy. CLEM analysis of infected HeLa LAMP1-GFP cells pulse-chased with HRP also revealed DAB product inside the outer lumen of double membrane SIF (type 2 SIT) and within the SCV at 8 h p.i. (Figure S10).

Next, we analyzed the early stage of *Salmonella* infection to test if DAB product localizes within leading SIF (LS). CLEM of HeLa cells 4 h p.i. revealed LAMP1-GFP-positive single membrane SIF (type 1 SIT or LS), completely filled with the DAB product (Figure S11). In conclusion, these results indicate that after the conversion of single membrane tubules (type 1 SIT, LS) to double membrane tubules (type 2 SIT, TS), the endocytosed content of LS eventually accumulates inside the outer lumen of TS.

### Formation of the double membrane SIF is dependent on the function of SPI2-T3SS effectors SseF and SseG

**Figure 8:**
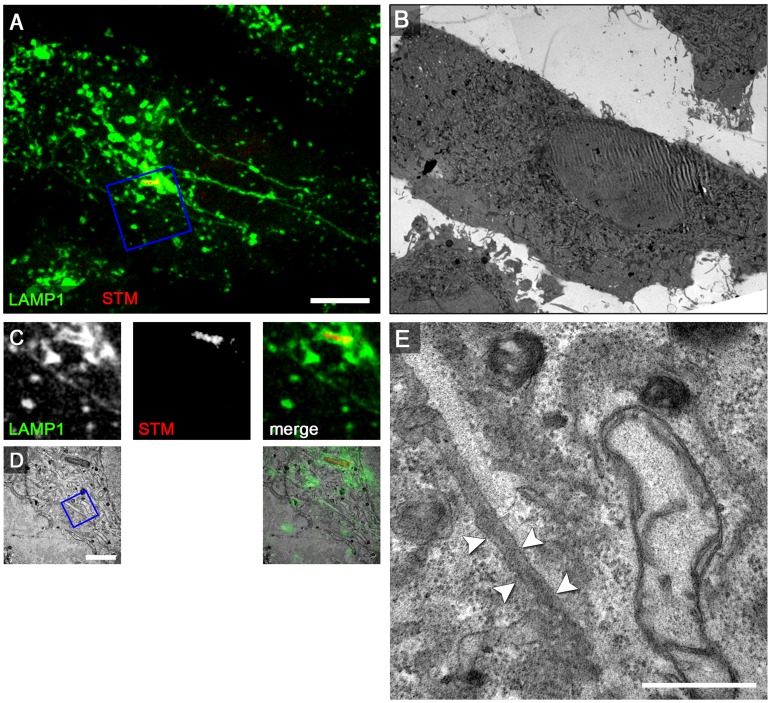
The SPI2-T3SS effector SseF is required for induction of double membrane SIF. For CLEM analysis, HeLa cells stably transfected with LAMP1-GFP (green) were seeded on a Petri dish with a gridded coverslip and infected with the *Salmonella sseF*-deficient strain expressing mCherry (STM, red). After live cell imaging at 8 h p.i. by CLSM (A, MIP) cells were fixed immediately on stage. *sseF*-infected HeLa cells exhibit thin LAMP1-positive tubules. B) Low magnification TEM micrograph of the same cell. Details for LAMP1-positive thin SIF and *Salmonella* within SCV are shown by correlative live cell CLSM (C, F single Z plane) and TEM (D, G) micrograph. E, H) Higher magnifications of a SIF. The single membrane tubule is indicated by arrowheads. A cell representative of two biological replicates is shown (1–2 technical replicates with each 2–4 cells). Scale bars: 10 µm (A, B), 2 µm (C, D, F, G), 500 nm (E, H).

We have previously reported that mutant strains deficient in SPI2-T3SS effector proteins SseF or SseG induce the formation of SIF with altered morphology [35], i.e. less intense labeling by fluid phase markers and LAMP1-GFP fluorescence, thinner appearance and higher susceptibility to fragmentation by chemical fixation with *para*-formaldehyde (PFA). PFA-fragmented SIF in *sseF* or *sseG*-infected HeLa cells are also known as pseudo-SIF [21]. We found that tubular membrane compartments induced by the *sseF* or *sseG* strain were maintained after fixation with glutaraldehyde (GA) used conventionally for TEM. Compared to PFA-fixation of *sseF*-infected HeLa cells with 67.6±5.1% pseudo-SIF formation, fixation by GA resulted in 4.3±2.1% pseudo-SIF and 95.7±2.1% continuous SIF, as judged by light microscopy (N = 100 cells, 3 replicates). However, SIF in *sseF*-infected cells appeared more heterogeneous in morphology in ultrastructure and were reminiscent of LS observed at early time points of infection (Figure 5). CLEM revealed that SIF in *sseF*-infected HeLa LAMP1-GFP cells at 8 h p.i. were delimited by a single membrane with electron-dense content (Figure 8), like type 1 SIT. The mean diameter of SIF in *sseF*-infected cells was 107±18 nm (N = 6 cells with 5 measurements per SIF). This diameter is less than a half of the mean diameter of the double membrane SIF (221±65 nm) but in the range of the diameter of single membrane SIF (120±46 nm, see above).

We next performed CLEM of *sseF*-infected HeLa LAMP1-GFP cells after pulse-chase labeling with BSA-Rhodamine followed by DAB photo-conversion. TEM analysis of SCV-derived membrane tubules in these cells revealed that electron-dense DAB product localizes inside the entire lumen of the single membrane SIF at 8 h p.i. (Figure S12). CLEM experiments with HRP showed a similar phenotype (data not shown). In cells infected with the *sseF*-deficient strain complemented with WT *sseF*, the SIF double membrane phenotype was restored (Figure S13). An *sseF* strain expressing episomal *sseF*_Δ200–205_ was previously reported to be unable to induce normal SIF [36] and we found that cells infected with *sseF* strain expressing *sseF*Δ_200–205_ contained only thinner single membrane SIF (Figure S14). Our previous work showed that a defect in *sseG* phenocopies the *sseF* mutation. CLEM analyses of cells infected with the *sseG*-deficient strain showed results similar to the *sseF* strain, since *sseG*-infected cells displayed single membrane SIF with electron-dense content (Figure S15).

The SPI2-T3SS effector protein SifA has a key role in virulence since a *sifA-*deficient strain is highly attenuated in systemic virulence and intracellular replication. The *sifA* strain fails to induce SIF and loses the SCV membrane during intracellular replication, thereby escaping into the cytoplasm [15]. Interestingly, Boucrot *et al*. [37] showed that a transient ectopic expression of SifA in epithelial cells leads to LAMP1-positive aggregations and also thin SIF-like tubular structures. We investigated the ultrastructure of SifA-induced SIF-like tubules in non-infected HeLa cells co-transfected with LAMP1-mCherry and GFP-SifA by CLSM and CLEM. CLSM indicated that 9.3±2.5% of co-transfected HeLa cells showed a SIF-like phenotype (Figure S16A), compared to 15±5% reported before [37]. Micrographs of the observed thin LAMP1-mCherry and GFP-SifA-positive SIF-like structures clearly show single membrane tubules (Figure S16B–E), comparable to type 1 SIT, but much thinner (41±10 nm). Thus, SifA appears to be sufficient for the formation of LAMP1-positive single membrane tubules in uninfected HeLa cells, but is not sufficient to induce double membrane tubules. In summary, our results show that the effector proteins SseF and SseG are not required for formation of single membrane SIF (type 1 SIT), but are essential for biogenesis of double membrane SIF (type 2 SIT), while ectopic expression of SifA is sufficient to induce single membrane tubules.

### The spatial organization of SCV and SIT

**Figure 9:**
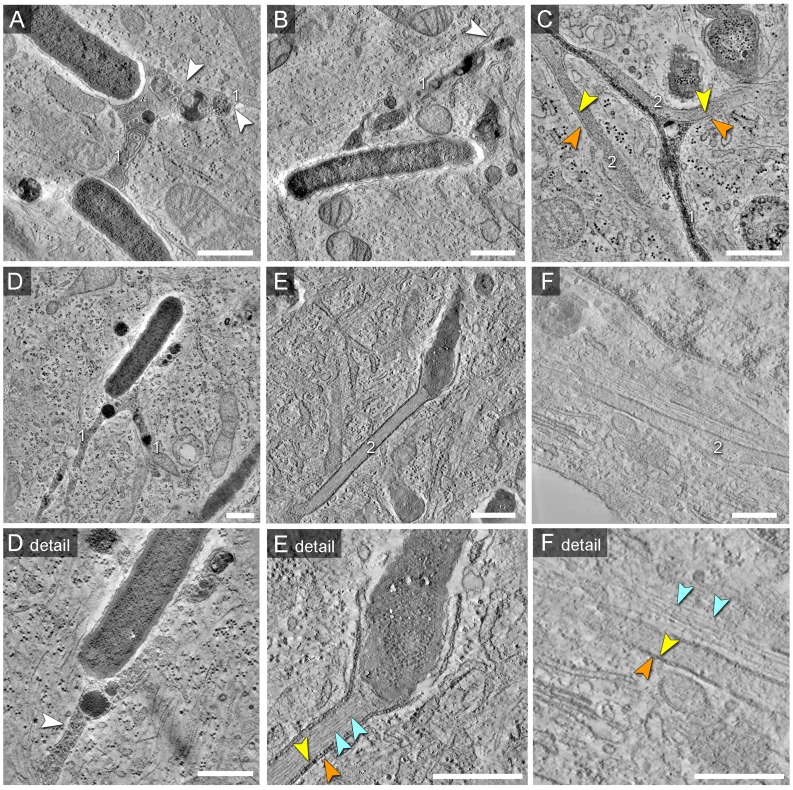
Morphology of SCV and SIT analyzed by EM tomography. HeLa cells were infected with *Salmonella* WT as before, fixed and processed for EM tomography of partial cell volumes. A) to F) shows representative images corresponding to the tilt series shown in Movies S2, S3, S4, S5, S6, S7, S8, S9, S10. Note the presence of double membrane SIT indicated by orange and yellow arrowheads. SIT that are delimited by single membranes and that contain multi-lamellar vesicles and dense granules typical for late endosomes and lysosomes are referred to as type 1 SIT (A, B, D), with white arrowheads indicating SIT membranes. SIT that appear delimited by double membranes and lack multi-vesicular membranes are referred to as type 2 SIT (E, F). C) shows a ‘hybrid’ SIT resulting from partial fusion of two intertwined type 1 and type 2 SIT. E) shows a type 2 SIT with an inner space filled by a bundle of actin-like filaments, while SIT in F) are associated with F-actin filaments adjacent to the SIT (light blue arrowheads). Scale bars: 500 nm.

Finally, we addressed the complexity of SCV and SIT membrane organization by ET to reveal the ultrastructure of these compartments in 3D. HeLa cells were infected with WT *Salmonella*, fixed 10 h p.i. and 300 nm sections were used to generate tilt series from −60° to +60°. This approach identified *Salmonella* inside SCV with connecting SIT (Figure 9A, B, D, E).

The 3D reconstruction of partial cell volumes revealed both types of SIT previously observed by TEM on ultrathin sections: (i) single-membrane delimited SIT with a lumen containing electron-dense granules and multi-lamellar vesicular structures (type 1 SIT) (Figure 9A, Movie S2, Figure 9B, Movie S3, Figure 9C, Figure 9D, Movie S5, Movie S6), and (ii) double-membrane delimited SIT with a hollow lumen containing cytoplasmic components (type 2 SIT) (Figure 9C, Movie S4, Figure 9E, Movie S7, Movie S8, Figure 9F, Movie S9, Movie S10). In addition to the complexity of membrane folding within lumen of SIF, tomograms also revealed structural continuity between distant portions of SIF which otherwise appeared as separate structures on ultrathin sections. Interestingly, we also found branched SCV-derived tubular membrane structures that appeared as intertwined type 1 and type 2 SIT (Figure 9C, Movie S4). This once more suggests that the two structurally distinct membrane tubules may represent different developmental stages, a process we described earlier as leading-to-trailing SIF phenotype during dynamic membrane remodeling in living cells [9], and in this study as a possible conversion of single membrane tubules (type 1 SIT, LS) to double membrane tubules (type 2 SIT, TS).

**Figure 10:**
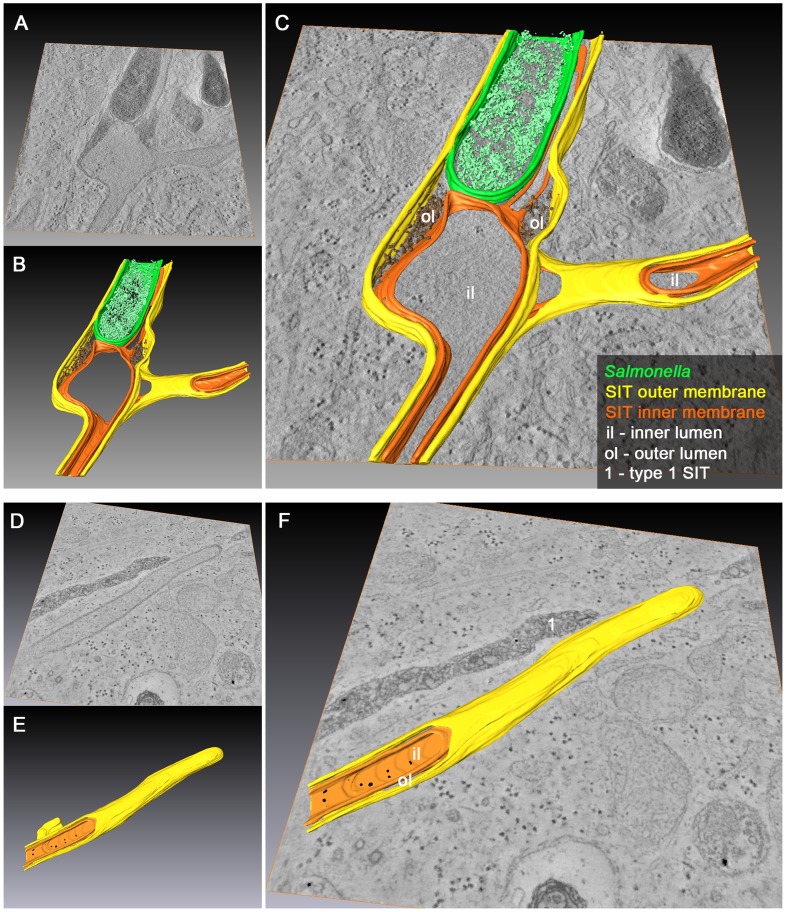
3D organization of SCV and SIT in *Salmonella*-infected cells. HeLa cells were infected with *Salmonella* WT for 10 h and processed for EM tomography of partial cell volumes followed by 3D-surface rendering. Representative images of single and double tilt series are shown in A) and D). A–C) Example of an SCV with extending and branching type 2 SIT showing dense luminal content between the two adjacent membranes at the base of SIT. D–F) Example of type 2 SIT with a closed tip and two membranes that wrap up portion of ribosome-containing cytosol. Note the presence of an adjacent type 1 SIT (without 3D-surface rendering) with a dense luminal content and delimited by a single membrane (E). The corresponding tilt series are shown in Movie S11 and Movie S13, respectively. B) and E) show 3D-surface rendering of the SIT and SCV membrane organization representing Movie S12 and Movie S14. Inner and outer SIT membranes are indicated in orange and yellow, respectively. The resulting inner and outer lumen of the double membrane tubular structure are labeled with iL and oL, respectively. Merged TEM images and rendered models are shown in C) and F).

Closer inspection of membrane arrangement within type 2 SIT emerging from the SCV revealed that both, inner and outer membrane, are in continuum and that *Salmonella*, together with other endosomal content resides between these two membranes (Figure 10A–C, Movie S11, Movie S12). Volume rendering of the distal end of the type 2 SIT disclosed that the inner membrane can enwrap portions of cytoplasm including electron-dense ribosomes. The tip of this SIT was captured within the data set of the tilt series and the 3D-rendering indicates that the tip of the captured SIT is closed (Figure 10D–F, Movie S13, Movie S14). These findings further support our hypothesis that the inner lumen of type 2 SIT, at a certain stage of SIT development, is in continuum with the cytoplasm before it is compartmentalized, while the space between the inner and outer membrane of these structures is the *bona fide* luminal space of SCV/SIT containing endocytosed cargo and bacteria.

## Discussion

To our knowledge, this is the first systematic ultrastructural analysis of the intracellular environment of *Salmonella* in host cells and the fine structure of host cell compartments modified by activities of intracellular *Salmonella*. Our approach used combinations of live cell imaging, TEM of ultrathin sections, EM tomography and cytochemistry to reveal various novel aspects of *Salmonella*-driven manipulation of the host cell endosomal system. We anticipate that current models for the intracellular lifestyle of *Salmonella* have to be revised due to the findings reported in this study.

The limit of spatial resolution in light microscopy does not allow to obtain sufficient details in the organization of SCV and SIF, as for instance the distinction of single, double or multiple membranes forming these compartments. Consequently, the interpretation of presence or absence of canonical markers of the endosomal maturation has to be made with care. For example, given that SIF are composed of two membranes, different forms of biogenesis could explain the presence of lysosomal glycoproteins in SIF membranes. The marker may be located i) in the inner and outer membrane (both membranes are derived from late endosomes or lysosomes), ii) only in the outer membrane (outer membrane derived from late endosomes or lysosomes, origin of inner membrane is different), or iii) only in the inner membrane (situation inverse to ii). The data shown in this communication clearly highlight the importance of ultrastructural analyses in addition to analyses by light microscopy.

Besides new insight into the complexity of SCV membrane arrangement, our study revealed for the first time two morphologically distinct types of *Salmonella*-induced tubules (SIT) in *Salmonella*-infected epithelial cells and macrophages: (i) type 1 SIT, delimited by a single membrane with a lumen containing electron-dense granules and vesicles, reminiscent of the luminal content of late endosomes, endolysosomes or in part lysosomes [16], and (ii) type 2 SIT, composed of two membranes with the inner lumen containing host cell cytosol and the outer lumen containing the bacteria and endocytosed material. CLEM showed that both type 1 and 2 SIT are dynamic, lgp-positive membranes, thus identical with the previously described *Salmonella*-induced filaments or SIF (Figure 4, Figure 5, Figure S11). Moreover, type 1 SIT were identified as the earlier described leading SIF (LS) and type 2 SIT as the appropriate trailing SIF (TS) (Figure 5). Thus, we suggest that dynamic conversion of leading to trailing SIF corresponds to the conversion of single membrane tubules (type 1 SIT) to double membrane tubules (type 2 SIT) and that type 1 SIT represent developmental precursors of type 2 SIT.

Double membrane compartments are typical for autophagosomes. Double membrane formation has been reported during the maturation of Coronaviruses [38], [39], or Hepatitis C virus [40] whereby an LC3-associated, but autophagy-independent recruitment of host membrane was considered. It is well known that autophagy targets *Salmonella* that has been released into host cytosol due to loss of SCV integrity [25], but we showed that double membranes of type 2 SIT are formed independently from autophagy.

**Figure 11:**
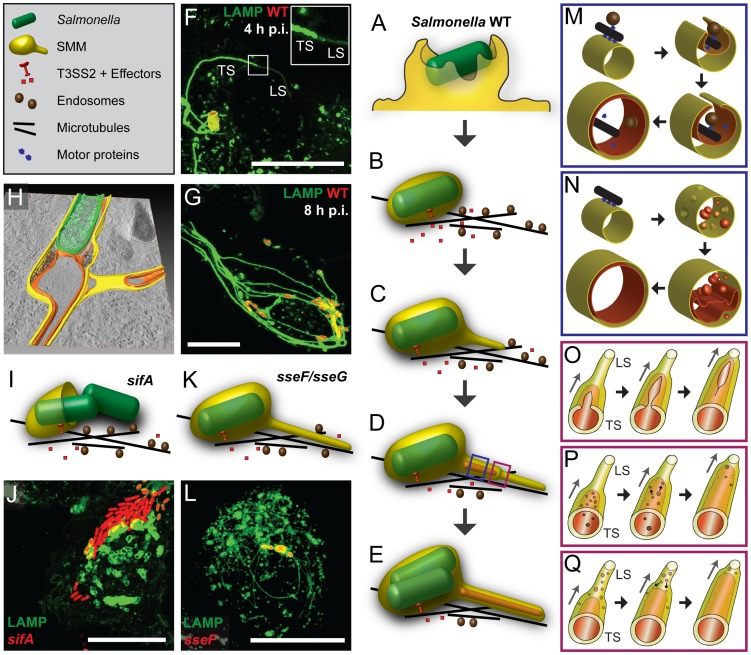
Models for the biogenesis of SIF. Schematic depiction of events leading to engulfment of cytosol and cytoskeletal elements, and formation of double membrane structures. After invasion of host cells by *Salmonella* involving ruffle formation (A), the internalized pathogen is located in a host-derived membrane compartment, called *Salmonella*-containing vacuole (SCV) that moves along microtubules (B) towards the MTOC. Subsequently, *Salmonella* activates the SPI2-T3SS and translocation of effector proteins into the host cytosol, thereby starting the induction of SIF. These tubular structures are formed through fusion processes with endosomes that move along microtubules (MT). In the initial phase (3–4 h p.i.) SIF consist only of one membrane (C) that undergo a process resulting in SIT consisting of double membranes (D, E). In the initial phase of centrifugal extension of SIT (D, F), we observed thinner single membrane SIF at the tips (leading SIF, LS) and thicker double membrane SIF at the base (trailing SIF, TS). At later time points (8–10 h p.i.) a stable double membrane SIF network is induced by *Salmonella* WT, accompanied by a strong reduction of host cell endosomes (E, G). The tomogram of a *Salmonella* WT-infected cell indicates the features of SCV and double membrane SIT (H). The *sifA* mutant strain is not capable of maintaining the membrane integrity of the SCV, *Salmonella* escape from the damaged SCV into the cytoplasm and no SIF formation is observed (I, J). The *sseF* and *sseG* mutant strains induce thin SIF consisting of one membrane (K, L). M) and N) show scenarios for conversion of single to double membrane SIF (blue boxes). M) Tubular membranes are formed along MT. By lateral extension, these membranes enwrap the guiding MT, as well as vesicles transported along MT and portions of cytoplasm. Membrane fusions results in formation of double membrane SIF. N) Alternatively, membrane invagination generates small vesicles that accumulate within single membrane SIF and the fusion of these vesicles results in formation of inner membrane tubule and its continuous extension. In both models, the lumen of the inner tubule is segregated from *Salmonella*. O), P), Q) Models for the dynamic conversion of LS to TS (red boxes). See main text for further details. Scale bars: 20 µm.

How can we explain the biogenesis of double membrane SIF with internal cytoskeletal elements? Figure 11 shows our working model with key events of SIF biogenesis. After invasion of host cells (Figure 11A), *Salmonella* within an early SCV start to translocate SPI2-T3SS effector proteins and to manipulate the host cell endocytic system (Figure 11B). We propose that single membrane SIF (type 1 SIT) emerge by tubulation of SCV membranes along cytoskeletal filaments (Figure 11C). This tubulation occurs longitudinal by fusion processes of endosomes and we and others previously reported the dynamic features of SIF in this initial phase of intracellular life of *Salmonella* [9], [10]. Our model implies that membrane extension also occurs laterally, resulting in the formation of a double membrane sheath enclosing microtubules, F-actin and portions of cytosol (Figure 11D, M). Since SIF cross sections and tomograms demonstrate continuity of SIF inner and outer membrane, we postulate that such a process ultimately results in membrane fusion and formation of double membrane SIF (type 2 SIT) (Figure 11E, M).

How does the transition from early, single membrane SIF (type 1 SIT, LS) to double membrane SIF (type 2 SIT, TS) occur? In light microscopy, the dynamic formation of thin LS with low LAMP1-GFP intensity, and bolder TS was observed [9], [10], see Figure 11F for an example. We think this observation is in line with a zipper-like mechanism of membrane fusion depicted in Figure 11O. The conversion into double membrane SIF is incomplete in the early stage of SIF formation, thus explaining the reversion of LS to TS and back. The limited resolution of light microscopy does not allow to trace the proposed zipper-like fusion events in living cells and the molecular mechanism underlying the membrane fusion has to be revealed by future work.

What is the origin of SIF membranes and luminal material? Previous studies indicated the interaction of the SCV with early and late endosomes [6], as well as with exocytic vesicles [7]. We demonstrated that SIF networks are in continuous interchange with endocytosed material, as observed for fluid tracers and fluorescent nanoparticles ([19], this study). The multiple interactions may result in a continuous network of SIF lumen with a very large volume connected to SCV (Figure 11G, H). Simple TEM micrographs in this study revealed electron-dense granules and vesicles for single membrane SIF, indicating a luminal content comparable to late endosomes, endolysosomes or, in part, lysosomes. CLEM experiments with HRP or BSA-Rhodamine demonstrated that the single membrane SIF lumen incorporates these fluid tracers and our model implies that, upon conversion into double membrane tubules, this luminal content represents the content of the outer lumen of double membrane SIF that is also in contact to *Salmonella* within the SCV. We propose that *Salmonella* within the SCV have a continuous interchange with endocytosed material such as medium components, but remain segregated from cytosolic components of the host cells that are entrapped in the inner SIF lumen of double membrane SIF (Figure 11H). This model clearly supports a role of SIF for the nutritional supply of *Salmonella* within the SCV.

The high abundance of lgp such as LAMP1 suggests a late endosomal/lysosomal origin of SIF membranes, however, the exposure of *Salmonella* within the SCV to antimicrobial effectors appears to be limited [4]. Recent work by the Holden group [41] demonstrated that by action of the effector protein SifA, intracellular *Salmonella* actively interfere with the proper activation and delivery of lysosomal hydrolases such as Cathepsin D to the SCV. Furthermore, there is evidence for distinct routes of delivery of lysosomal membrane material including lgp (‘LAMP carriers’) and the lysosomal hydrolases to late endosomes (‘MPR carriers’) [42]. By selectively recruiting ‘LAMP carriers’ and/or avoidance of ‘MPR carriers’, intracellular *Salmonella* would be able to generate extensive lgp-positive membrane compartments without exposure to lysosomal hydrolases. A further explanation could be the dilution of antimicrobial effectors in a large volume of SCV continual with volume of extensive network of SIF (Figure 3, [19]). _ENREF_16Luminal content of vesicles fusing with the SCV or SIF could be rapidly mixed with the luminal content of SIT, resulting in decreased concentrations of antimicrobial activities. Indeed, we also observed a decreased acidification of SCV if connections to SIF were present (YZ, MH, unpublished observations). This model may also explain the previous controversial observation of low degree of delivery of lysosomal enzymes to the SCV [4]. We clearly detect delivery of fluid tracers (NP, BSA-Rhodamine, HRP, etc.) to SCV and SIF. However, rapid dilution of incoming tracers within the luminal space of the network may explain why prolonged exposures (longer pulse times) are required to obtain detectable signals.

Which molecular mechanisms lead to fusion events resulting in double membrane SIF? Induction of SIF is dependent on function of the SPI2-T3SS and a subset of effector proteins, predominantly SifA, SseF, SseG, SopD2 and PipB2. Most likely, host cell-intrinsic mechanisms are manipulated by activities of SPI2-T3SS effectors. The interaction of SifA with SKIP is an excellent example [41], [43], [44]. SifA is essential for the induction of SIT and *sifA* mutant strains are defective in maintenance of the SCV (Figure 11I, J). Expression of GFP-SifA in uninfected epithelial cells leads to LAMP1-positive aggregations and also thin SIF-like tubular structures [37]. Here, we showed by CLEM that these SIF-like tubular structures represent single membrane tubules (Figure S16). Therefore, we propose that the SPI2-T3SS effector SifA could be sufficient for the formation of single membrane SIF. Yet action of SifA is insufficient to induce double membrane SIF. The exact mechanism of membrane fusion and longitudinal tubulation caused by SifA has to be examined in future work. Interestingly, mutant strains lacking SseF and SseG are attenuated in intracellular replication [35]. In *sseF*-and *sseG*-infected HeLa cells only thin SIF are formed (Figure 11K, L) and our CLEM analysis revealed single membrane SIF for both mutants (Figure 8, Figure S12, Figure S15). Consequently, we propose that SseF and SseG are the key SPI2-T3SS effectors involved in the lateral tubulation of single membrane SIF and the final membrane fusion leading to double membrane SIF. For SseF and SseG, the host cell targets appear less clear, the target candidates for SseF and SseG [45], [46] do not indicate involvement in membrane fusion. The molecular mechanism behind SseF-and SseG-mediated membrane remodeling still remains open, but our previous study [47] suggested that SseF is involved in Dynein recruitment to the SCV membrane, what is likely essential for extension of SIF from SCV along microtubules. The absence of double membrane SIF after infection with *sseF*- or *sseG*-deficient *Salmonella* may also be explained by a missing direct interaction of these effectors after membrane insertion. SseF is an integral protein of host endosomes [36] and a direct interaction with SseG has been demonstrated [48]. This model would imply that *Salmonella*, by means of the SPI2-T3SS, transfers an autonomous membrane fusion machinery into the host cell in order to induce extensive membrane aggregations resulting in double membrane compartments. Such novel function of T3SS effectors will require detailed experimental investigation.

Alternative models for biogenesis of SIF are shown in Figure 11N, P, Q. An inner tubule may also be formed by fusion of invaginated membrane vesicles located within the single membrane SIF (Figure 11N, Q). We observed some single membrane SIF with a multivesicular content (Figure 1B, Figure 8A–D). However, we neither observed invagination processes nor intermediate phases of fusion processes of the intraluminal vesicles inside single membrane SIF. Furthermore, the model proposed in Figure 11N, Q fails to explain mechanistically the enclosure of microtubules and F-actin, and we consider this model as less likely. Alternatively, vesicles moving along microtubules together with single membrane SIF could be entrapped the same way as cytoskeleton during the double membrane formation and afterwards the inner tubule could grow due to fusion events of the vesicular content with the SIF inner membrane from the inside (Figure 11M, P). Such double membrane SIF with vesicles inside the inner lumen were also observed in micrographs for early time points of *Salmonella* infection (Figure S7). At later time points after infection, vesicles were seldom observed inside the SIF inner lumen, indicating their disappearance, probably due to fusion with the inner membrane. It is also possible that events depicted in Figure 11O and P occur simultaneously, since both originate from lateral tubulation (Figure 11M).

The data reported here give rise to a number of further questions and experimental challenges regarding the intracellular lifestyle of *Salmonella*. The most important question is: How could intracellular *Salmonella* benefit from the formation of a double membrane SIF network? We consider three consecutive scenarios: i) By means of the SPI2-T3SS effector SifA intracellular *Salmonella* induce the formation of single membrane tubules extending from the SCV. This extensive tubular SIF network may generate a compartment in which incoming endocytic cargo accumulates and bactericidal lysosomal content is diluted. Such mechanism would provide nutrients for *Salmonella* within the SCV, but also decrease the local concentration of antimicrobial activities. For the dilution effect vesicles with various types of luminal content have to fuse to the SIF network. ii) Transport of both, endocytic and exocytic vesicles towards SCV and SIF and eventual fusion is critical for the successful intracellular lifestyle of *Salmonella*. A large number of fusion events may result in excessive amounts of membrane material at the SCV/SIF and dramatically increase their surface area. Here we speculate that a shortage of solid content within the volume of SCV/SIF causes insufficient physical support to maintain their circular profile and, together with a surplus of membrane, leads to a structural collapse of these membrane compartments. This hypothesis is supported by our findings that SCV membranes often fold into double-membrane curved sheets and invaginate or extend into SIF tubules (examples in Figure 1C, Figure 10C). Our model suggests how single membrane SIF (type 1 SIT) can extend both, longitudinally and laterally along microtubules and eventually wrap up and close to form double membrane SIF (type 2 SIT). It remains to be clarified to which extent and how this membrane rearrangement is controlled by *Salmonella*, e.g. through the action of SPI2-T3SS effectors SseF and SseG. iii) The formation of double membrane SIF through the entrapment of cytosol and especially the entrapment of cytoskeletal filaments into double membrane SIF will result in a stabilized tubular network. Within double membrane SIF, F-actin and microtubules are segregated from exchange with cytosolic components. The previously observed phenotype of microtubule bundling in *Salmonella*-infected cells may be explained by accumulation of microtubules inside double membrane SIF [14]. Furthermore, the accumulation of cytoskeleton within double membrane SIF could explain the previous observation of decreasing dynamics of SIF at later time points after infection [9], [10]. The stabilized SIF network would allow the SCV to maintain the juxtanuclear, Golgi-associated position that is essential for efficient intracellular proliferation [5], [47], [ reviewed in 49]. A stabilized SIF network would ensure the supply of *Salmonella* within the SCV with endocytosed nutrients and probably nutrients delivered by fusion events of distinct vesicles.

Finally, the morphology and function of other SIT like SIST and LNT remains an open question. In our study performed on HeLa or RAW264.7 cells at early (4–5 h) or late (8–12 h) time points p.i., all tubular structures connected to SCV observed were LAMP1-GFP-positive. For SIST, formation at very late time points after infection of HeLa cells was described [11], thus our analyses most likely did not cover SIST formation. The highest number of LNT was reported for the infection of HeLa cells with a *sifA sopD2* double mutant strain [12], which was not analyzed here. Future work has to reveal the details of the complex tubular membrane compartments induced by intracellular *Salmonella*.

## Materials and Methods

### Bacterial strains and growth conditions

*Salmonella enterica* serovar Typhimurium strain NCTC12023 was used as wild-type strain and isogenic mutant strains used in this study are listed in Table 2 [15], [35], [50]. For complementation, the *sseF* mutant strain was used harboring P*_sseA_ sscB sseF*::HA or P*_sseA_ sscB sseF*_Δ200–205_::HA [36]. If required for detection in live cell imaging, the strains harbored pFPV25.1 [51], pFPV-mCherry/2 [10] or pETcoco1-PrpsM-mCherry2 [52] for constitutive expression of GFP or mCherry, respectively. Bacteria were grown in LB broth at 37°C with aeration.

**Table 2:** Bacterial strains and plasmids used in this study.

Designation	relevant genotype	Reference, GenBank ID
*S. enterica* serotype Typhimurium strains		
NCTC12023	wild type	lab collection
P2D6	*ssaV*::mTn*5*	[50]
P3H3	*sifA*::mTn*5*	[15]
HH107	Δ*sseF*::*aphT*	[54]
HH108	Δ*sseG*::*aphT*	[54]
Plasmids		
pEGFP-C1		Clontech
pmCherry-C1		Clontech
pFPV25.1		[51]
pFPV-mCherry/2		[10]
p3589	pETcoco1 const. mCherry	[52]
p3432	LAMP1-eGFP	[9]
p3451	LAMP1-mCherry	this study
pBABEpuro GFP-LC3	GFP-LC3b	[55], Addgene 22405, U05784
pmCherry-ATG5	ATG5	[33], Addgene 13095, AB048349
pmCherry-ATG5-K130R	ATG5-K130R	[33], Addgene 13096
p3942	GFP::*sifA* in pEGFP	this study
p2643	P*_sseA_ sscB sseF*::HA	[36]
p3349	P*_sseA_ sscB sseF*_Δ200–205_::HA	[36]

### Cell lines and cell culture

For all experiments the non-polarized epithelial cell line HeLa (American Type Culture Collection, ATCC no. CCL-2) was used. HeLa cells were cultured in Dulbecco's modified Eagle's medium (DMEM) containing 4.5 g×l^−1^ glucose, 4 mM stable glutamine and sodium pyruvate (Biochrom) and supplemented with 10% inactivated fetal calf serum (iFCS) (Sigma-Aldrich) at 37°C in an atmosphere containing 5% CO_2_ and 90% humidity. The Lentivirus-transfected stable HeLa cell line expressing LAMP1-GFP was cultured under same conditions.

The murine macrophage-like cell line RAW264.7 (ATCC no. TIB-71) stably transfected with LAMP1-GFP via Lentivirus transfection was cultured in DMEM containing 4.5 g×l^−1^ glucose and 4 mM stable glutamine supplemented with 6% iFCS at 37°C in an atmosphere containing 5% CO_2_ and 90% humidity. For activation of RAW264.7 cells 7.5 ng×ml^−1^ IFNγ (BD Heidelberg) was added to the cell culture medium 24 h before infection. Prior to infection the cells were provided with fresh medium without IFNγ.

### HeLa cells transfection

HeLa or HeLa LAMP1-GFP cells were cultured for one day in various culture vessels depending on the experimental setup and transfected with FUGENE HD reagent (Promega) according to manufacturer's instruction. In brief, 0.5–2 µg of plasmid DNA were solved in 25–100 µl DMEM without iFCS and mixed with 1–4 µl FUGENE reagent (ratio of 1∶2 for DNA to FUGENE). After 10 min incubation at room temperature (RT) the transfection mix was added to the cells in DMEM with 10% iFCS for at least 18 h. Before infection the cells were provided with fresh medium without transfection mix.

### Host cell infection

For infection of HeLa or HeLa LAMP1-GFP cells, *Salmonella* strains were grown in LB broth overnight (ON), diluted 1∶31 in fresh LB and subcultured for 3.5 h in order to induce maximal SPI1-dependent invasion. The infection of HeLa cells was performed at different multiplicities of infection (MOI) for 25 min. RAW264.7 LAMP1-GFP cells were infected with ON cultures of *Salmonella* strains for 25 min. Subsequently, cells were washed thrice with PBS and incubated for 1 h with medium containing 100 µg×ml^−1^ gentamicin (Applichem) to kill non-invaded bacteria. Finally the medium was replaced by medium containing 10 µg×ml^−1^ gentamicin for the rest of the experiment.

### Fluid-phase marker pulse-chase and DAB conversion

Fluid phase markers Gold-BSA-Rhodamine and BSA-Rhodamine were synthesized as described before [19] and applied to the cells after *Salmonella* infection at various time points prior to imaging. Co-localization analyses were performed using Leica LAS AF software (Leica, Wetzlar, Germany). As positive control, HeLa cells simultaneously pulse-chased with Dextran-Alexa Fluor 488 (Invitrogen) and Dextran-Alexa Fluor 568 (Invitrogen) were used. The same threshold was applied to each dataset for LAMP1-GFP and Gold-BSA-Rhodamine to calculate co-localization rates and Pearson's correlation coefficient (Imaris, Bitplane).

For 3,3′-diaminobenzidine tetrahydrochloride (DAB, Sigma) as TEM marker, cells were pulse-chased for indicated periods of time with 400 µg×ml^−1^ BSA-Rhodamine or 10 mg×ml^−1^ horseradish peroxidase (HRP, Type IV, Sigma) in complete DMEM medium. After live cell imaging of a region of interest (ROI) by CLSM, the cells were fixed and blocked as described in ‘Sample preparation for CLEM’. For the DAB conversion fixed cells were covered with freshly-prepared ice-cold 1 mg×ml^−1^ DAB in 0.2 M HEPES buffer. For BSA-Rhodamine, the ROI was viewed again by CLSM and DAB photo-conversion was started by irritating the ROI with blue light (Xenon lamp, full power) until a brown DAB polymer was visible by eye. For HRP the DAB conversion was initialized by adding H_2_O_2_ to a final concentration of 0.01% for 2 min in the dark. After DAB conversion in both cases, the DAB solution was removed and the cells were washed several times in HEPES buffer. The HRP-fed cells were checked by light microscopy for DAB conversion. Subsequently, the samples were further processed for TEM as described in ‘Sample preparation for CLEM’.

### Live cell imaging

For live cell imaging DMEM was replaced by imaging-medium consisting of Minimal Essential Medium (MEM) with Earle's salts, without NaHCO_3_, without L-glutamine and without phenol red (Biochrom) supplemented with 30 mM HEPES (4-(2-hydroxyethyl)-1-piperazineethanesulfonic acid) (Sigma-Aldrich), pH 7.4. The imaging studies were performed using the confocal laser-scanning microscope (CLSM) Leica SP5 equipped with an incubation chamber maintaining 37°C and humidity during live cell imaging. The software LAS AF (Leica, Wetzlar, Germany) was used for setting adjustment, image acquisition and image processing. At various time points post infection images were acquired with the 100× objective (HCX PL APO CS 100×) (Leica, Wetzlar, Germany) and the polychroic mirror TD 488/543/633 for the three channels GFP and mCherry/Rhodamine and DIC. A permanent LAMP1-GFP expressing HeLa cell line was generated by lentiviral transfection as described in Text S1. We confirmed that intracellular phenotypes of *Salmonella* were identical to those observed in transiently LAMP1-GFP infected HeLa cells.

### Analysis of autophagy

HeLa cells (2.5×10^4^) were seeded in eight well chamber slides (Ibidi), allowed to adhere overnight, and subsequently transfected with plasmid GFP-LC3b (Addgene, pBABEpuro GFP-LC3). The next day, cells were infected with WT *Salmonella* at an MOI of 100. Gold- Rhodamine nanoparticles were pulsed at 3 h p.i. for 1 h in order to indicate the *Salmonella*-induced tubular structures. Living cells were imaged at various time points post infection to track the co-localization of GFP-LC3b with *Salmonella* or *Salmonella-*induced tubular structures. As control for induction of autophagy by starvation, transfected HeLa cells were washed thrice with PBS and incubated in PBS at 37°C for 1 h.

### Sample preparation for CLEM

HeLa LAMP1-GFP cells (1×10^5^) or RAW264.7 LAMP1-GFP cells (1.5×10^5^) were seeded in a petri dish with a gridded coverslip (MatTek, Ashland, MA) two days prior to the infection with *Salmonella*. One day before infection HeLa cells were transfected, if necessary, and RAW264.7 cells were activated by IFNγ. After the infection with an MOI of 75, if required, cells were pulse-chased with fluid-phase markers for indicated periods of time post infection. At indicated time points after infection, a ROI was observed by live cell imaging and cells were fixed as fast as possible directly on stage with pre-warmed 2.5% glutaraldehyde (Electron Microscopy Sciences) in buffer (0.2 M HEPES, pH 7.4, 5 mM CaCl_2_) for 1 h at 37°C. After rinsing the cells several times in buffer, unreacted glutaraldehyde was blocked by 50 mM glycine in buffer for 15 min, followed by rinses in buffer. Post-fixation was performed with 2% osmium tetroxide (Electron Microscopy Sciences) in buffer containing 1.5% potassium ferricyanide (Sigma) and 0.1% ruthenium red (Applichem) for 1 h at 4°C in the dark. After several washing steps the cells were dehydrated in a cold graded ethanol series and finally one rinse in anhydrous ethanol and two rinses in anhydrous acetone at room temperature. The gridded coverslip was removed from the Petri dish and cells were infiltrated and flat-embedded in mixes of acetone and EPON812 (Serva). During the removal of the gridded coverslip from the polymerized EPON block the engraved coordinates were transferred to the EPON surface and allowed trimming around the ROI. Serial 70 nm sections were cut with an ultramicrotome (Leica EM UC6) and collected on formvar-coated EM copper grids. After staining with uranyl acetate and lead citrate, cells were observed with TEM (Zeiss EFTEM 902 A), operated at 80 kV and equipped with a 2K wide-angle slow-scan CCD camera (TRS, Moorenwies, Germany). Images were taken with the software ImageSP (TRS image SysProg, Moorenwies, Germany). For image analysis, software packages LAS AF (Leica. Wetzlar), ImageJ (http://rsbweb.nih.gov/ij/) and Imaris (Bitplane, Zürich) were used. Stitching and overlay of CLSM and TEM images was done using Photoshop 5.5 (Adobe).

### High pressure freezing and freeze substitution

For superior membrane preservation, samples were processed by high pressure freezing-freeze substitution (HPF-FS). Cells were grown in glass-bottom dishes (MaTek Corp.) on top of sapphire discs with a carbon finder-grid mask and coated with poly-L-lysine. At appropriate time post infection with MOI of 100, the dishes were processed directly for HPF as follows: Discs were removed from media, placed between hexadecane-treated aluminum specimen carriers with a 0.1 mm cavity and immediately transferred to LEICA HP010 holder and processed by HPF. Discs were then transferred from liquid nitrogen to the cryovials containing freeze-substitution medium (1% OsO_4_ and 0.2% uranyl acetate in Acetone) and then to FS device (LEICA AFS1) for FS processing with final embedding in EPON.

### EM and EM tomography

Thin-sections were post-stained with 2% lead citrate in water and examined using a Morgagni electron microscope (FEI). Thick sections (300 nm) of HPF-FS samples were placed on a slot grid covered with a formvar film and decorated with 10 nm protein-A gold particles on both sides for image alignment. Grids were placed in a high-tilt holder (Fischione Model 2020) and dual-axis ET were carried out using a Tecnai F30 (FEI) electron microscope (operated at 300 kV) equipped with a field emission gun and a 4084×4084 pixels CCD camera (Eagle, FEI). Tomographic tilt ranges were typically from +60° to −60° with an angular increment of 1° pixel size ranging from 0.5 to 1 nm. Alignments, 3D reconstructions, and merging of serial tomograms were done with IMOD software suite [53]. The volume segmentations were performed with the Amira 4.1 visualization package (Visage Imaging, Berlin, Germany).

## Supporting Information

Figure S1 **Complex organization of host cell membranes in** ***Salmonella*** **-infected HeLa cells.** HeLa cells were infected with *Salmonella* WT and subjected to HPF-FS 10 h p.i. Overview (A, B) and detail (C, D) TEM micrographs show SCV and connected SIT. In B and D, inner (orange) and outer (yellow) SIT membranes are indicated. Red and light red arrowhead indicate the outer and inner membrane of *Salmonella* (S), respectively. Scale bars: 2 µm (A, B), 500 nm (C, D).
(TIF)

Figure S2 **Dimensions of SIT.** Representative examples of longitudinal sections (A) and cross sections (B) through double membrane SIT observed in *Salmonella*-infected HeLa cells. Images were used for determination of SIT dimensions. Scale bars: 100 nm. C) Quantification of diameters of single and double membranes SIT. Means and standard deviations of 35 and 150 diameters for single and double membrane SIT, respectively. Statistical significance was determined by Student's t test and is indicated as ***, *p*<0.001.
(TIF)

Figure S3 **Intracellular replication of** ***Salmonella*** **in HeLa-LAMP1-GFP cells.** Parental HeLa cells and the clones 6–9, 6–10, 7–11 and 7–20 stably expressing hLAMP1-GFP were infected with *Salmonella* WT and *ssaV* strains each harboring pFPV25/mCherry for 2 and 16 h. The number of intracellular bacteria was determined by plating lysates and determination of colony-forming units (cfu). The x-fold replication was calculated by dividing cfu at 16 h p.i. by cfu at 2 h p.i.
(TIF)

Figure S4 **Co-localization of immuno-stained LAMP1 and LAMP1-GFP (green) in stably transfected HeLa cell clone P6–9.** Cells from clone P6–9 were cultured and infected with *Salmonella* WT expressing mCherry (red). At 16 h p.i., the cells were fixed and stained with mouse α hLAMP1, coupled with rabbit α mouse Alexa Fluor 568. CLSM images show 3D projections of a Z stack. Scale bar: 20 µm.
(TIF)

Figure S5 **SIF dynamics in clone P6–9 HeLa cells stably expressing LAMP1-GFP (green).** Clone P6–9 was infected with *Salmonella* WT expressing mCherry (red). About 5 h p.i., time lapse images were acquired over a period of 8 min using a CLSM. The images displayed are MIP of the Z stacks and the corresponding movie is shown in Movie S1. Note the appearance of leading SIF (LS) and trailing SIF (TS) White and yellow arrowheads indicate representative TS and LS, respectively. Time stamp, min∶sec. Scale bar: 10 µm.
(TIF)

Figure S6 **Organization of the endosomal system in non-infected HeLa cells.** HeLa cells expressing LAMP1-GFP (green) were seeded in a Petri dish with gridded coverslip. On the next day cells were pulsed-chased with BSA-Rhodamine (red) for 3 h and fluorescence images of living cells were acquired by CLSM after chase of 2 h (A, D, two different examples) Cells were fixed immediately on stage and prepared for CLEM as described in Experimental Procedures. B, E) Stitched TEM images of cells shown in A and D (above) with high magnification of ROI (indicated by blue boxes) below (C, F). Note the presence of large numbers of spherical, LAMP1-positive compartments with luminal BSA-Rhodamine and the absence of extensive tubular compartments. Cells representative of two biological replicates are shown (1–2 technical replicates with 2–4 cells each). Scale bars: 10 µm (A, B, D, E), 2 µm (C, F).
(TIF)

Figure S7 **Early-stage SIF in HeLa cells showing double membrane SIT with internal vesicles.** The experimental set-up was as described for Figure 5. Panel F) shows details of *Salmonella* (S) within SCV connected to a double membrane SIF shown in E). Note the presence of numerous vesicles (V) within the double membrane SIF. A cell representative for three biological replicates is shown (1–3 technical replicates with 2–4 cells each). Scale bars: 10 µm (A, B), 1 µm (C, D), 500 nm (E, F).
(TIF)

Figure S8 **Single membrane tubules in uninfected and** ***Salmonella*** **-infected RAW264.7 macrophages.** RAW264.7 cells stably expressing LAMP1-GFP (green) were seeded in Petri dishes with a gridded coverslip and kept uninfected (left panel) or were infected with *Salmonella* WT expressing mCherry (STM, red) (right panel). Live cell imaging was performed (8 h p.i. for infected cells) to visualize LAMP1-GFP-positive structures (A, F, MIP; C, H single Z plane). Subsequently, the cells were fixed and processed for CLEM to reveal the ultrastructure. Several low magnification images were stitched to visualize the cell morphology (B, G). Higher magnification images were used to align LM and TEM images (C, D; H, I). Details of LAMP1-GFP-positive single membrane tubules in an uninfected cell (E) and LAMP1-GFP-positive single membrane SIF in a *Salmonella* infected cell (J) are shown. Note the presence of intraluminal vesicles in both kinds of tubules. Representative cells of two biological replicates are shown (1–3 technical replicates with 2–4 cells each). Scale bars: 10 µm (A, F), 2 µm (C, D, H, I), 500 nm (E, J).
(TIF)

Figure S9 **Host cell autophagy targets a subpopulation of intracellular** ***S. enterica*** **, but is not responsible for the double membrane formation of** ***Salmonella*** **-induced tubular structures.** A) HeLa cells were transfected with GFP-LC3b (green), infected with *Salmonella* WT expressing mCherry (STM, red), and living cells were imaged by CLSM at indicated time points. At 3 h p.i., cells were pulsed with Gold-BSA-Rhodamine nanoparticles (red) for 1 h in order to label SCV and SIT (in merge at 4 h, 8 h p.i.). A subpopulation of intracellular *Salmonella* was targeted by GFP-LC3b. No co-localization of labeled SIT with GFP-LC3b was observed. B–F) Analyses of ATG5-inhibited cells. HeLa cells expressing LAMP1-GFP (green) were transfected with pmCherry-ATG5 or pmCherry-ATG5-K130R (red) and infected with *Salmonella* WT expressing mCherry (STM, red). At 8 h p.i. living cells were subjected to quantification of SIF (B), or CLEM analysis of SIF ultrastructure (C–F). B) Transfected HeLa cells were compared to non-transfected cells and SIF formation was quantified (100 cells analyzed for each of three biological replicates, statistical analysis was performed by one-way ANOVA versus WT and significances are indicated as follows: * = *p*<0.05, n.s. = not significant). SIF formation in HeLa cells transfected with pmCherry-ATG5-K130R is reduced. C–F) CLEM of HeLa cells transfected with pmCherry-ATG5-K130R. ATG5-K130R expressing cells showed no obvious changes in morphology as determined by light microscopy (C, D) and TEM (E, F). Note the double membrane structure for LAMP1-positive SIF (F). A cell representative of two biological replicates is shown (1–3 technical replicates with 2–3 cells each). Scale bars: 5 µm (A), 10 µm (B), 1 µm (D, E), 500 nm (F).
(TIF)

Figure S10 **The outer lumen of double membrane SIF is accessible to endocytosed material.** A) Scheme of the experiment. HeLa cells expressing LAMP1-GFP (green) were seeded on a petri dish with a gridded coverslip. Cells were infected with *Salmonella* WT expressing mCherry (STM, red). HRP was added as fluid tracer to the medium 2–5 h p.i. At 8 h p.i., selected cells were imaged by light microscopy (B, CLSM, MIP) and immediately fixed on stage. DAB conversion by HRP was performed and cells were prepared for TEM. Several images of the same section were stitched for an overview (C). Details of LAMP1-positive SIF are shown on fluorescence images (D, G, single Z plane), and TEM micrographs (E, F, H, I). I) Note DAB deposition between the two adjacent membranes of SIT (F) and within lumen of the SCV in direct contact with bacteria (I). A cell representative of four biological replicates is shown (1–3 technical replicates with 2–4 cells each). Scale bars: 10 µm (B, C), 1 µm (D, E, G, I, J, K), 500 nm (F, G).
(TIF)

Figure S11 **Early-stage SIF in HeLa cells are in interchange with endocytosed material.** The experimental set-up was as described for Figure S10, but with HRP pulse/chase for 3 h and DAB conversion by HRP after on-stage fixation. Panel E) shows the presence of the DAB polymer within the whole lumen of single membrane SIF. A cell representative for two biological replicates is shown (1–2 technical replicates with 2–4 cells each). Scale bars: 10 µm (A, B), 2 µm (C, D), 500 nm (E).
(TIF)

Figure S12 **The SPI2-T3SS effector SseF is required for induction of double membrane SIT.** HeLa cells expressing LAMP1-GFP (green) were seeded on a petri dish with a gridded coverslip. After infection with the *Salmonella sseF*-deficient strain expressing mCherry (STM, red), cells were pulse-chased with BSA-Rhodamine 2–5 h p.i. After live cell imaging at 8 h p.i. by CLSM (A, MIP), cells were fixed immediately on stage. Finally, DAB photo-conversion by Rhodamine was performed and samples were prepared for TEM. A) *sseF*-infected HeLa cells exhibit thin LAMP1-positive, BSA-Rhodamine-positive tubules. B) TEM micrograph of the same cell. CLEM of two different magnified ROIs showing only SIF (C, D, single Z plane) and *Salmonella* within SCV (F, G, single Z plane). E, H) Magnifications of structures of interest. Tubular structure with single membrane and DAB polymer inside the whole tubule lumen (E). *Salmonella* inside SCV with DAB polymer inside SCV (H). A cell representative for two biological replicates is shown (1–3 technical replicates with 2–3 cells each). Scale bars: 10 µm (A), 2 µm (D, G), 1 µm (E).
(TIF)

Figure S13 **Complementation of the** ***sseF*** **strain with WT** ***sseF*** **restores induction of double membrane SIF.** Infection and imaging was performed as for Figure 8, but the *sseF* mutant strain complemented with WT *sseF* expressing mCherry (STM, red) was used. The complementation of *sseF* leads to LAMP1-positive double membrane tubule in infected HeLa cells. Live cell imaging at 8 h p.i. (A, MIP of CLSM), low magnification TEM (B), details (C, single Z plane of CSLM; D, TEM) and higher magnification TEM (E) of a SIF. A cell representative for two biological replicates is shown (1–2 technical replicates with 2–4 cells each). Scale bars: 10 µm (A, B), 1 µm (C, D), 500 nm.
(TIF)

Figure S14 **Complementation of the** ***sseF*** **strain with** ***sseF*** **_Δ200–205_ fails to restore double membrane SIF formation.** Infection and imaging was performed as for Figure 8, but the *sseF* mutant strain complemented with *sseF*_Δ200–205_ expressing mCherry (STM, red) was used. The Δ*sseF* strain expressing *sseF*_Δ200–205_ leads to LAMP1-positive single membrane tubules (white arrowheads) in infected HeLa cells. Live cell imaging at 8 h p.i. (A, MIP of CLSM), low magnification TEM (B), details (C, single Z plane of CSLM; D, TEM) and higher magnification TEM (E) of a SIT. A cell representative of two biological replicates is shown (1–2 technical replicates with 2–4 cells each). Scale bars: 10 µm (A, B), 1 µm (C, D), 500 nm (E).
(TIF)

Figure S15 **The SPI2-T3SS effector SseG is required for induction of double membrane SIF.** Infection and imaging was performed as for Figure 8, but the *sseG* mutant strain expressing mCherry (STM, red) was used. HeLa cells infected with the Δ*sseG* strain exhibit thin LAMP1-positive tubules composed of a single membrane. Live cell imaging at 8 h p.i. (A, MIP of CLSM), low magnification TEM (B), details (C, single Z plane of CSLM; D, TEM) and higher magnification TEM (E) of a SIF. A cell representative of two biological replicates is shown (1–2 technical replicates with 2–3 cells each). Scale bars: 10 µm (A, B), 2 µm (C, D), 500 nm (E).
(TIF)

Figure S16 **Expression of SPI2-T3SS effector** ***sifA*** **in HeLa cells leads to formation of SIF-like LAMP1-positive single membrane tubules.** A) HeLa cells were transfected with pEGFP-SifA (green), the control vector pEGFP, transfection reagent FuGene only, or mock transfected. The next day, pulse-chase with Dextran-Alexa 568 for 3 h (red) was performed and for each condition at least 100 living cells were scored for presence of SIF-like tubules. Three biological replicates were performed, and statistical significances between pEGFP-SifA-transfected cells and other treatments were calculated by one-way ANOVA, and are indicated by *** = *p*<0.001, n.d. = not detectable. B–E) HeLa cells were cotransfected with pEGFP-SifA (green) and pLAMP1-mCherry (red) and subjected to CLEM the next day. After live cell imaging by CLSM (B, MIP) cells were immediately fixed on stage. HeLa cells showed many very thin tubular structures positive for EGFP-SifA and LAMP1-mCherry. Details of tubules are shown by correlative live cell CLSM (C, single Z plane) and TEM (D) micrographs. E) Higher magnifications of a network of tubules. Note the single membrane structure of the very thin tubules (arrowheads). A cell representative of three technical replicates with 2–4 cells each is shown. Scale bars: 10 µm (B), 1 µm (C, D), 500 nm (E).
(TIF)

Movie S1 **Dynamics of SIF in HeLa cells constitutively expressing LAMP1-GFP.** Time lapse sequence corresponding to Figure S5. The data set is shown as tilted 3D projection using Imaris.
(MP4)

Movie S2 **ET tilt series of a** ***Salmonella*** **-infected HeLa cell.** The movie corresponds to the still image of a single tilt position shown in Figure 9A.
(MP4)

Movie S3 **ET tilt series of a** ***Salmonella*** **-infected HeLa cell.** The movie corresponds to the still image of a single tilt position shown in Figure 9B.
(MP4)

Movie S4 **ET tilt series of a** ***Salmonella*** **-infected HeLa cell.** The movie corresponds to the still image of a single tilt position shown in Figure 9C.
(MP4)

Movie S5 **ET tilt series of a** ***Salmonella*** **-infected HeLa cell.** The movie corresponds to the still image of a single tilt position shown in Figure 9D.
(MP4)

Movie S6 **ET tilt series of a** ***Salmonella*** **-infected HeLa cell.** The movie corresponds to the still image of a single tilt position shown in Figure 9D detail.
(MP4)

Movie S7 **ET tilt series of a** ***Salmonella*** **-infected HeLa cell.** The movie corresponds to the still image of a single tilt position shown in Figure 9E.
(MP4)

Movie S8 **ET tilt series of a** ***Salmonella*** **-infected HeLa cell.** The movie corresponds to the still image of a single tilt position shown in Figure 9E detail.
(MP4)

Movie S9 **ET tilt series of a** ***Salmonella*** **-infected HeLa cell.** The movie corresponds to the still image of a single tilt position shown in Figure 9F.
(MP4)

Movie S10 **ET tilt series of a** ***Salmonella*** **-infected HeLa cell.** The movie corresponds to the still image of a single tilt position shown in Figure 9F detail.
(MP4)

Movie S11 **ET tilt series of double membrane SIF.** The SIF are connected to the SCV as shown in Figure 10A.
(MP4)

Movie S12 **Volume rendering and color annotations for SIF.** SIF inner membrane (yellow), SIF outer membrane (orange), and *Salmonella* (green) corresponding to Movie S11 and Figure 10B.
(MP4)

Movie S13 **EM tilt series of a peripheral part of a double membrane SIF.** The movie corresponds to Figure 10D.
(MP4)

Movie S14 **Volume rendering and color annotations for SIF.** SIF inner membrane (yellow) and SIF outer membrane (orange) corresponding to Movie S13 and Figure 10E.
(MP4)

Text S1 **Stable cell line generation.**
(DOCX)
