# Supplementary figures and images for: Reorganization of the Endosomal System in Salmonella-Infected Cells: The Ultrastructure of Salmonella-Induced Tubular Compartments
Source: PLoS Pathog. 2014 Sep 25;10(9):e1004374. doi: 10.1371/journal.ppat.1004374 (PMC4177991; doi:10.1371/journal.ppat.1004374)

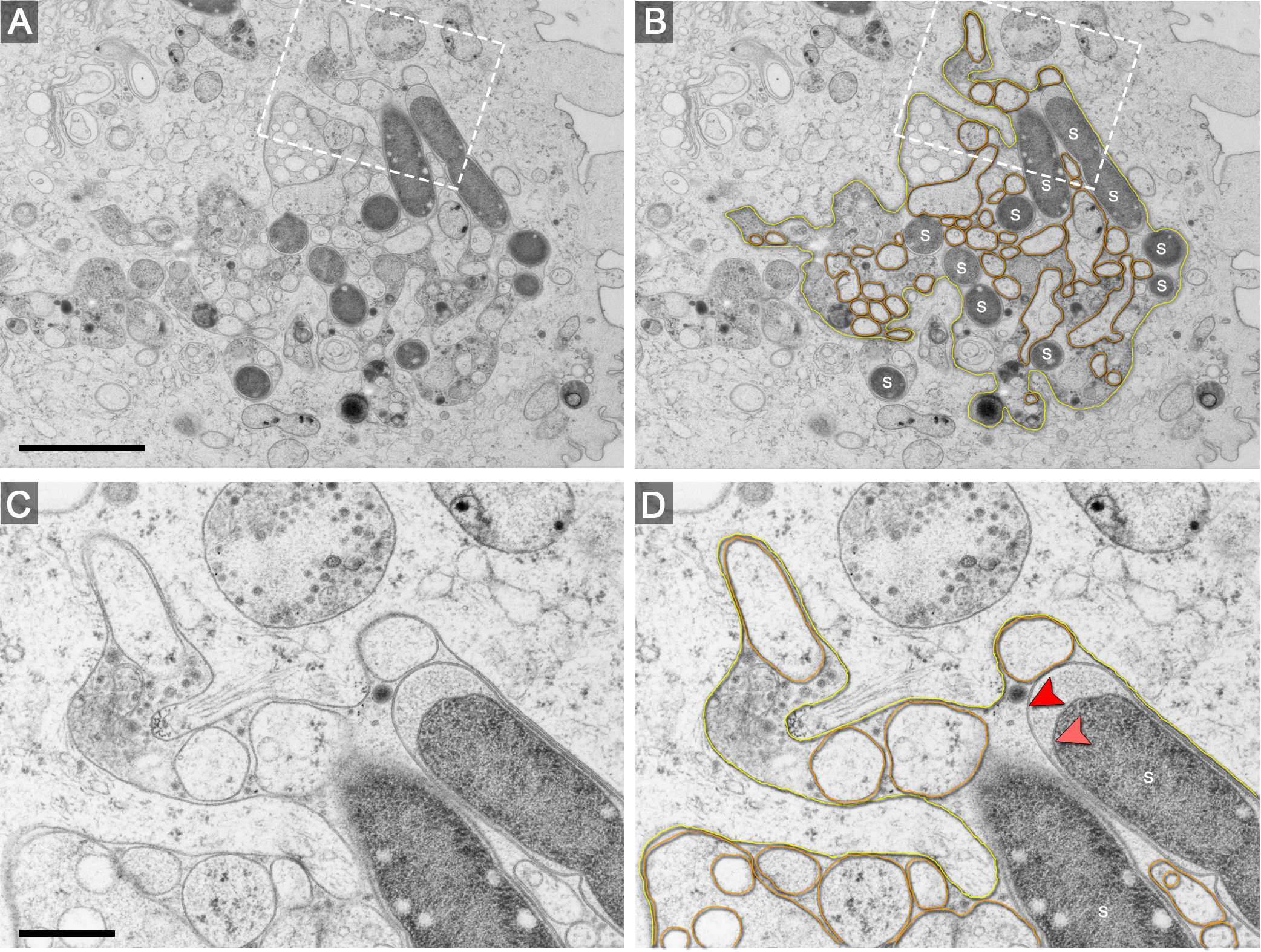

Supplement: Figure S1 — Complex organization of host cell membranes in Salmonella -infected HeLa cells. HeLa cells were infected with Salmonella WT and subjected to HPF-FS 10 h p.i. Overview (A, B) and detail (C, D) TEM micrographs show SCV and connected SIT. In B and D, inner (orange) and outer (yellow) SIT membranes are indicated. Red and light red arrowhead indicate the outer and inner membrane of Salmonella (S), respectively. Scale bars: 2 µm (A, B), 500 nm (C, D). (TIF) [file ppat.1004374.s001.tif]

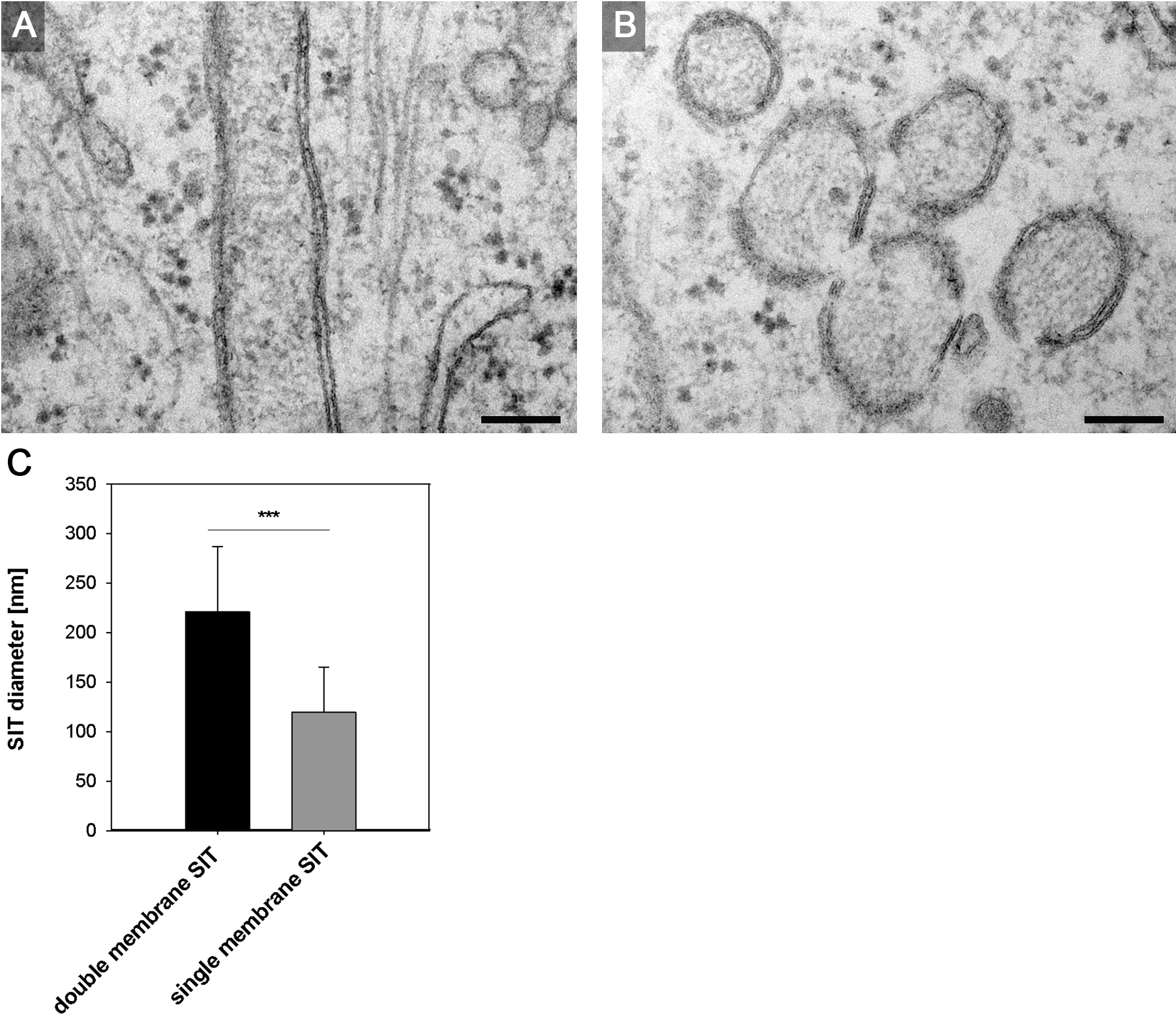

Supplement: Figure S2 — Dimensions of SIT. Representative examples of longitudinal sections (A) and cross sections (B) through double membrane SIT observed in Salmonella-infected HeLa cells. Images were used for determination of SIT dimensions. Scale bars: 100 nm. C) Quantification of diameters of single and double membranes SIT. Means and standard deviations of 35 and 150 diameters for single and double membrane SIT, respectively. Statistical significance was determined by Student's t test and is indicated as ***, p<0.001. (TIF) [file ppat.1004374.s002.tif]

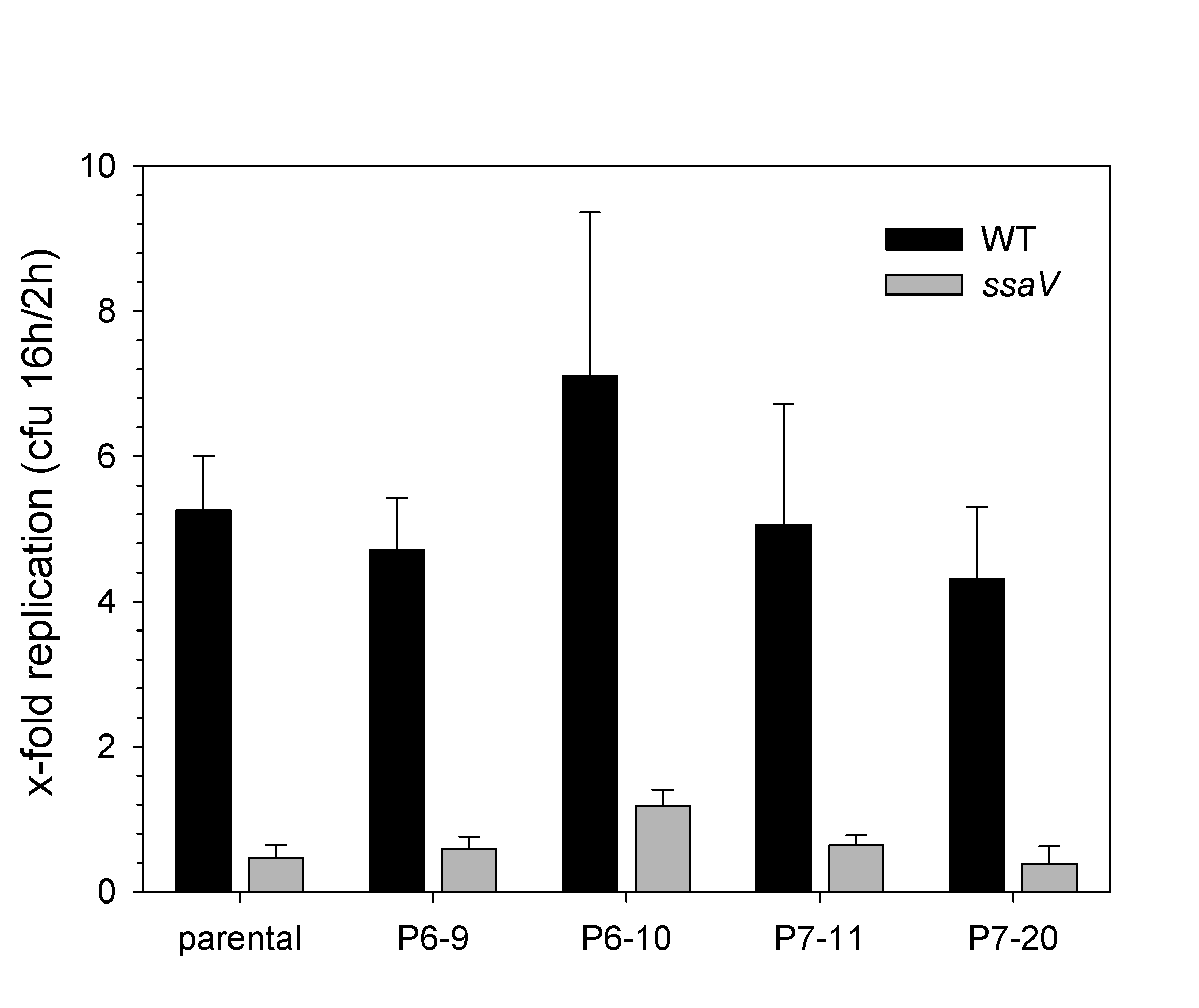

Supplement: Figure S3 — Intracellular replication of Salmonella in HeLa-LAMP1-GFP cells. Parental HeLa cells and the clones 6–9, 6–10, 7–11 and 7–20 stably expressing hLAMP1-GFP were infected with Salmonella WT and ssaV strains each harboring pFPV25/mCherry for 2 and 16 h. The number of intracellular bacteria was determined by plating lysates and determination of colony-forming units (cfu). The x-fold replication was calculated by dividing cfu at 16 h p.i. by cfu at 2 h p.i. (TIF) [file ppat.1004374.s003.tif]

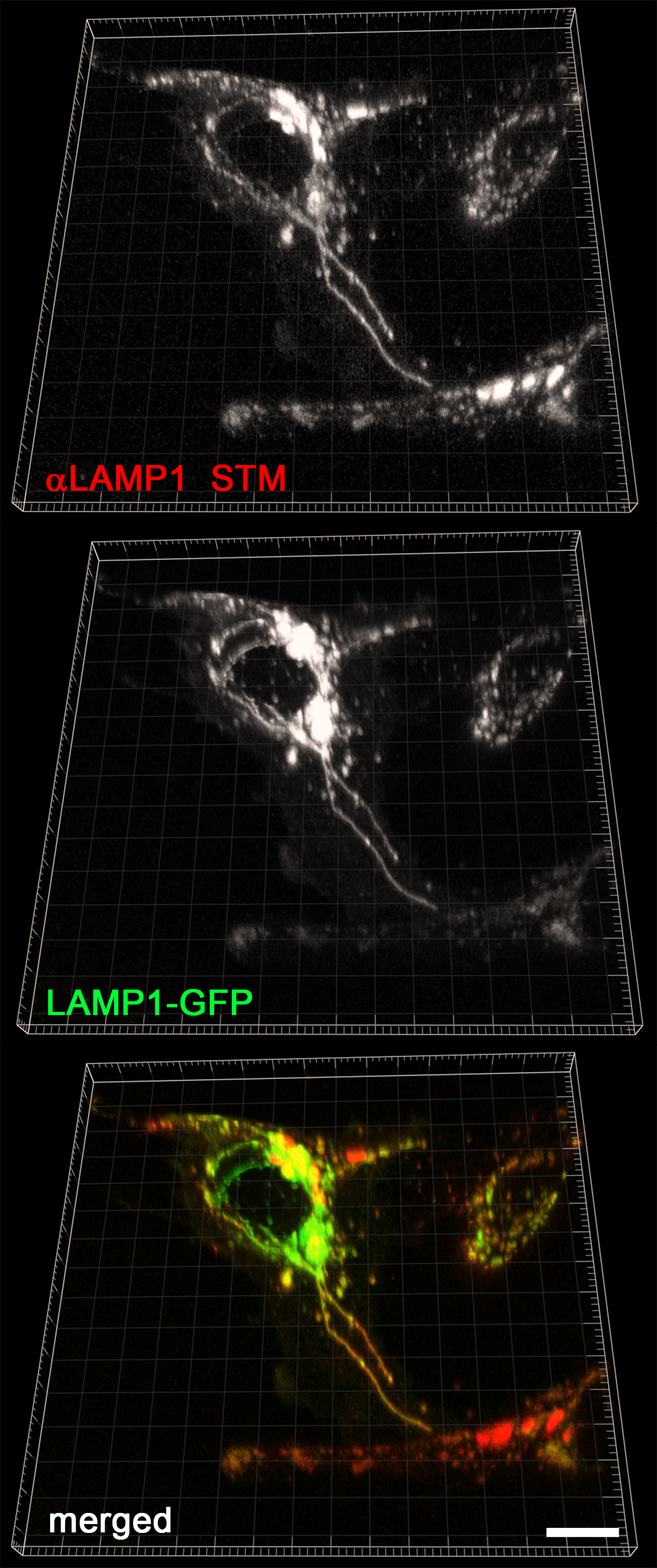

Supplement: Figure S4 — Co-localization of immuno-stained LAMP1 and LAMP1-GFP (green) in stably transfected HeLa cell clone P6–9. Cells from clone P6–9 were cultured and infected with Salmonella WT expressing mCherry (red). At 16 h p.i., the cells were fixed and stained with mouse α hLAMP1, coupled with rabbit α mouse Alexa Fluor 568. CLSM images show 3D projections of a Z stack. Scale bar: 20 µm. (TIF) [file ppat.1004374.s004.tif]

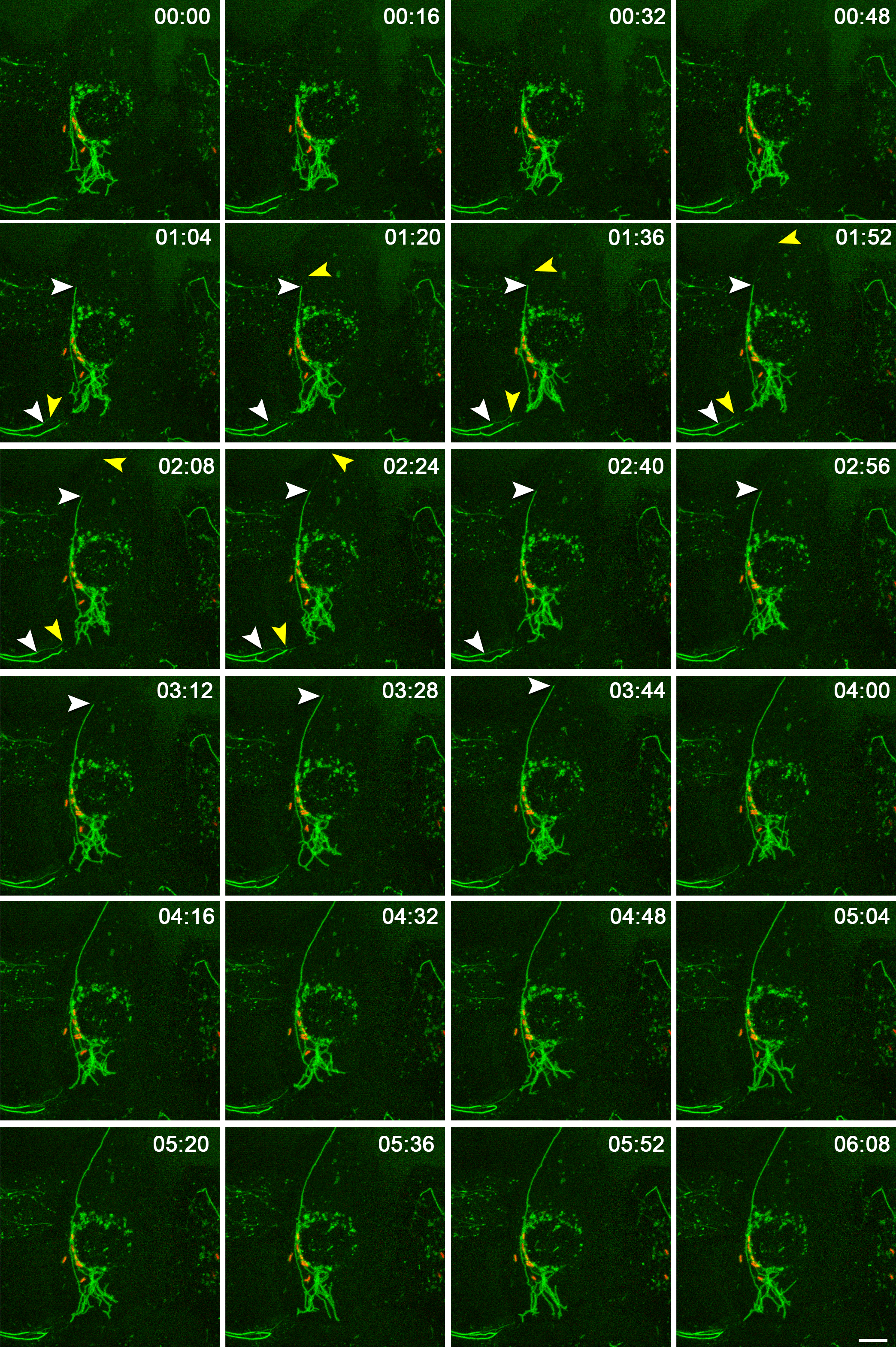

Supplement: Figure S5 — SIF dynamics in clone P6–9 HeLa cells stably expressing LAMP1-GFP (green). Clone P6–9 was infected with Salmonella WT expressing mCherry (red). About 5 h p.i., time lapse images were acquired over a period of 8 min using a CLSM. The images displayed are MIP of the Z stacks and the corresponding movie is shown in Movie S1. Note the appearance of leading SIF (LS) and trailing SIF (TS) White and yellow arrowheads indicate representative TS and LS, respectively. Time stamp, min∶sec. Scale bar: 10 µm. (TIF) [file ppat.1004374.s005.tif]

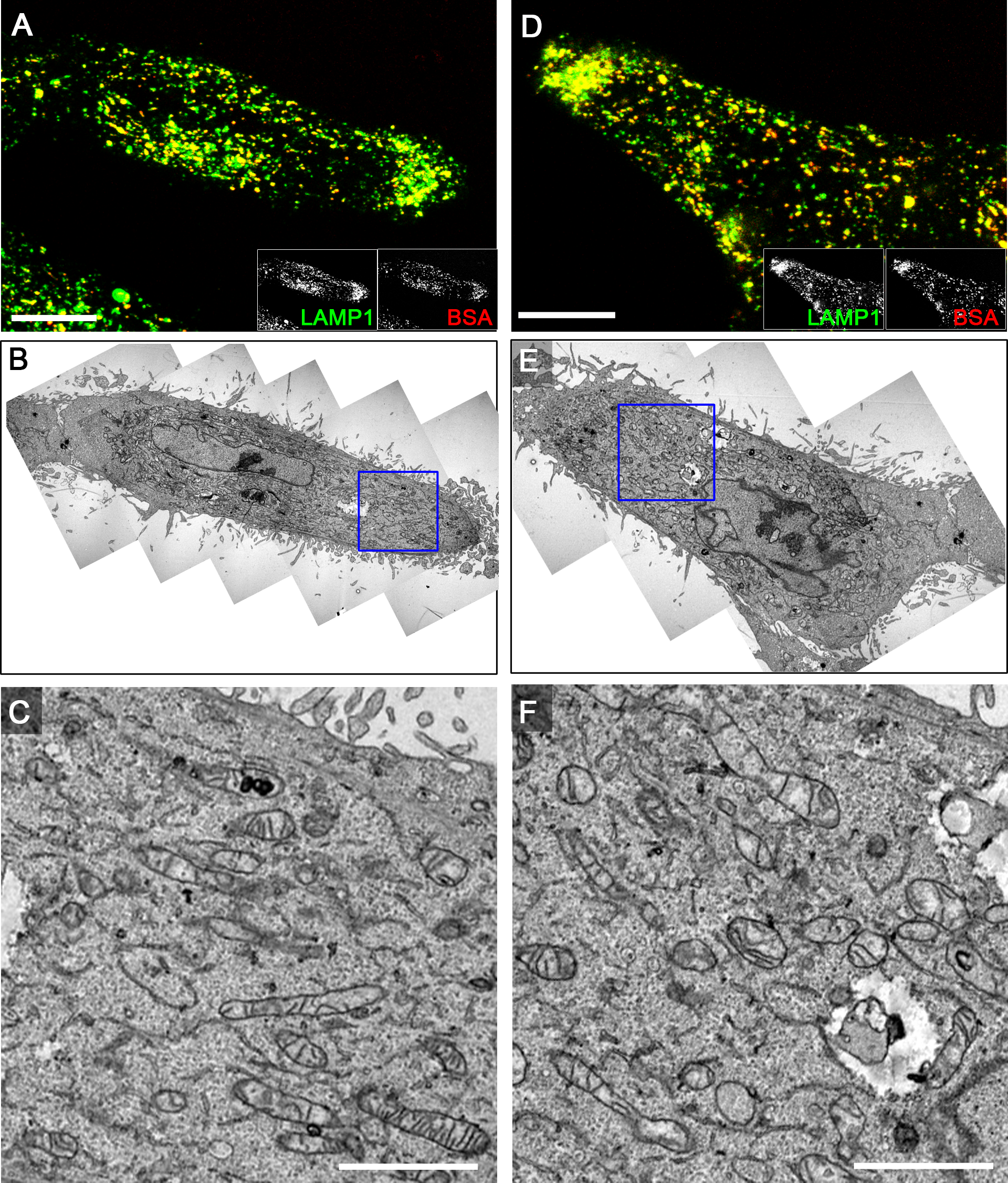

Supplement: Figure S6 — Organization of the endosomal system in non-infected HeLa cells. HeLa cells expressing LAMP1-GFP (green) were seeded in a Petri dish with gridded coverslip. On the next day cells were pulsed-chased with BSA-Rhodamine (red) for 3 h and fluorescence images of living cells were acquired by CLSM after chase of 2 h (A, D, two different examples) Cells were fixed immediately on stage and prepared for CLEM as described in Experimental Procedures. B, E) Stitched TEM images of cells shown in A and D (above) with high magnification of ROI (indicated by blue boxes) below (C, F). Note the presence of large numbers of spherical, LAMP1-positive compartments with luminal BSA-Rhodamine and the absence of extensive tubular compartments. Cells representative of two biological replicates are shown (1–2 technical replicates with 2–4 cells each). Scale bars: 10 µm (A, B, D, E), 2 µm (C, F). (TIF) [file ppat.1004374.s006.tif]

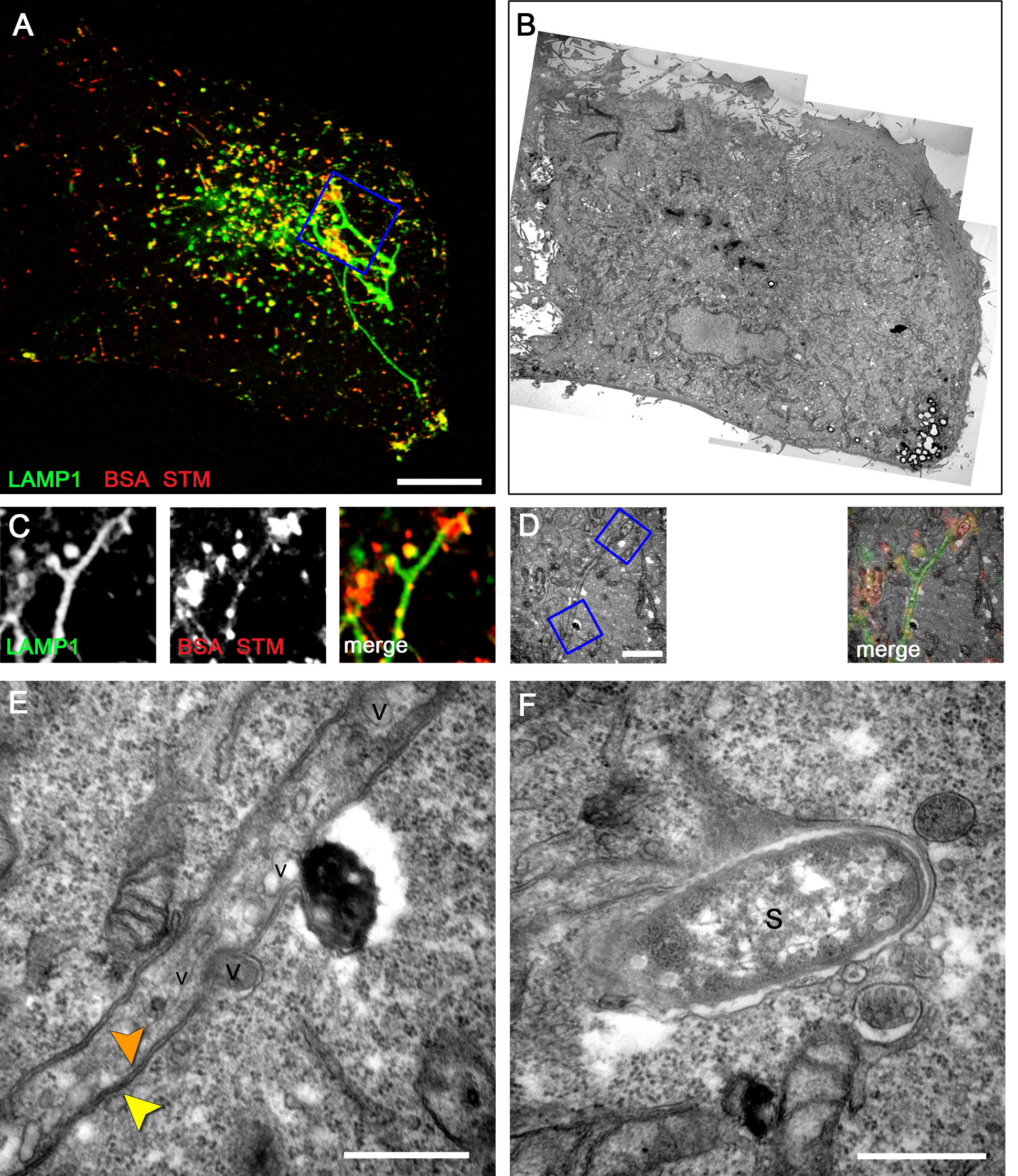

Supplement: Figure S7 — Early-stage SIF in HeLa cells showing double membrane SIT with internal vesicles. The experimental set-up was as described for Figure 5. Panel F) shows details of Salmonella (S) within SCV connected to a double membrane SIF shown in E). Note the presence of numerous vesicles (V) within the double membrane SIF. A cell representative for three biological replicates is shown (1–3 technical replicates with 2–4 cells each). Scale bars: 10 µm (A, B), 1 µm (C, D), 500 nm (E, F). (TIF) [file ppat.1004374.s007.tif]

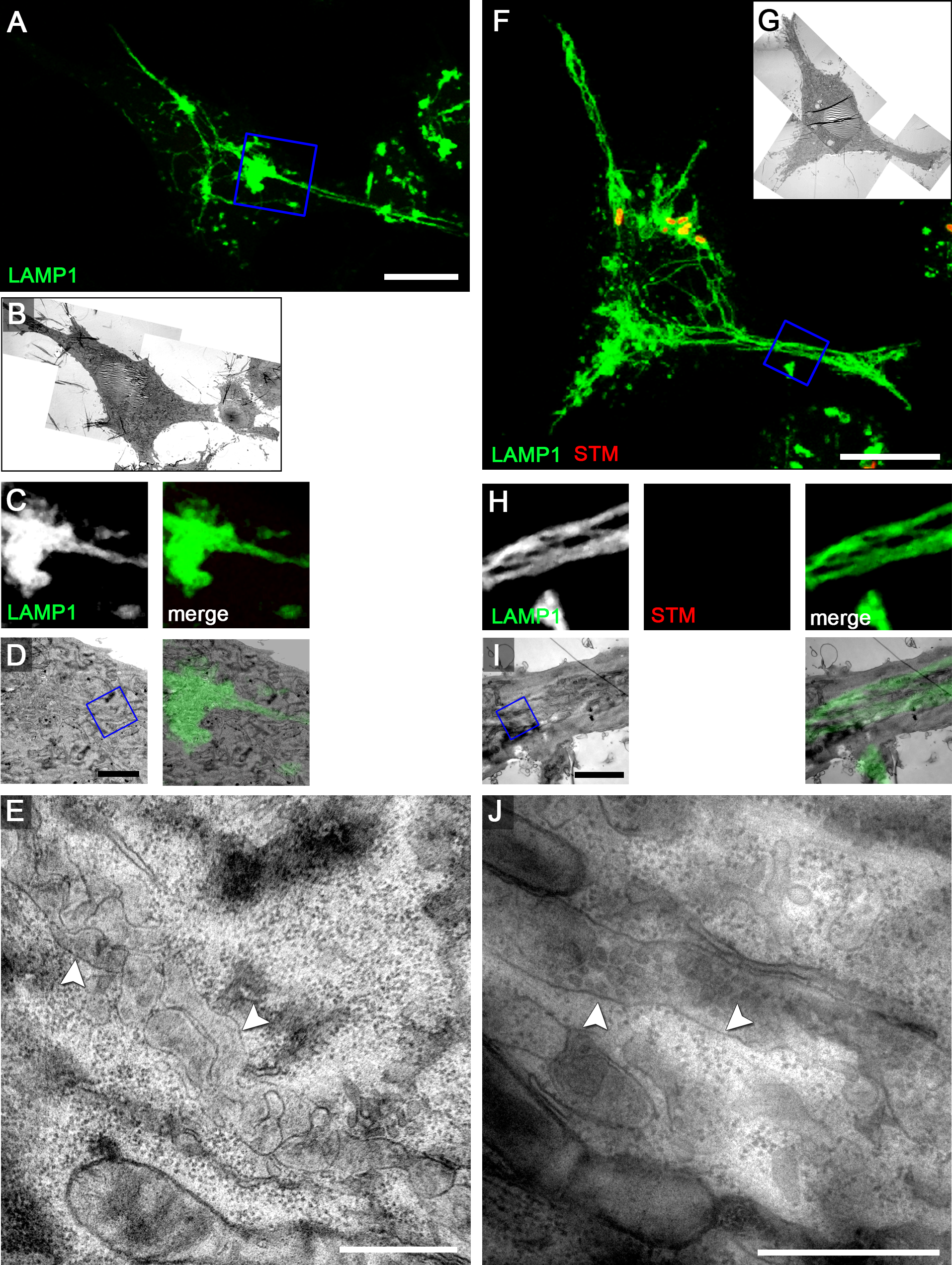

Supplement: Figure S8 — Single membrane tubules in uninfected and Salmonella -infected RAW264.7 macrophages. RAW264.7 cells stably expressing LAMP1-GFP (green) were seeded in Petri dishes with a gridded coverslip and kept uninfected (left panel) or were infected with Salmonella WT expressing mCherry (STM, red) (right panel). Live cell imaging was performed (8 h p.i. for infected cells) to visualize LAMP1-GFP-positive structures (A, F, MIP; C, H single Z plane). Subsequently, the cells were fixed and processed for CLEM to reveal the ultrastructure. Several low magnification images were stitched to visualize the cell morphology (B, G). Higher magnification images were used to align LM and TEM images (C, D; H, I). Details of LAMP1-GFP-positive single membrane tubules in an uninfected cell (E) and LAMP1-GFP-positive single membrane SIF in a Salmonella infected cell (J) are shown. Note the presence of intraluminal vesicles in both kinds of tubules. Representative cells of two biological replicates are shown (1–3 technical replicates with 2–4 cells each). Scale bars: 10 µm (A, F), 2 µm (C, D, H, I), 500 nm (E, J). (TIF) [file ppat.1004374.s008.tif]

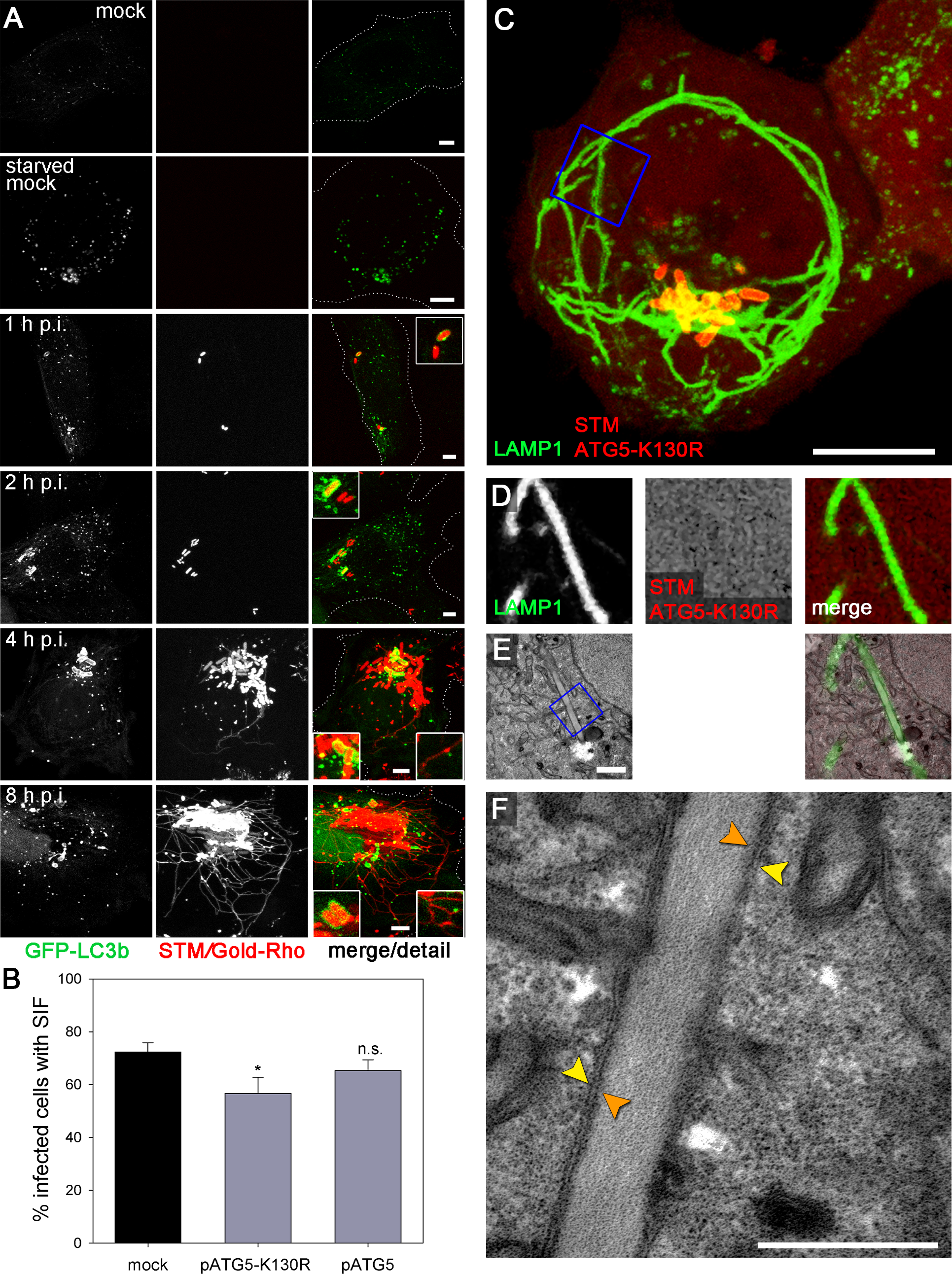

Supplement: Figure S9 — Host cell autophagy targets a subpopulation of intracellular S. enterica , but is not responsible for the double membrane formation of Salmonella -induced tubular structures. A) HeLa cells were transfected with GFP-LC3b (green), infected with Salmonella WT expressing mCherry (STM, red), and living cells were imaged by CLSM at indicated time points. At 3 h p.i., cells were pulsed with Gold-BSA-Rhodamine nanoparticles (red) for 1 h in order to label SCV and SIT (in merge at 4 h, 8 h p.i.). A subpopulation of intracellular Salmonella was targeted by GFP-LC3b. No co-localization of labeled SIT with GFP-LC3b was observed. B–F) Analyses of ATG5-inhibited cells. HeLa cells expressing LAMP1-GFP (green) were transfected with pmCherry-ATG5 or pmCherry-ATG5-K130R (red) and infected with Salmonella WT expressing mCherry (STM, red). At 8 h p.i. living cells were subjected to quantification of SIF (B), or CLEM analysis of SIF ultrastructure (C–F). B) Transfected HeLa cells were compared to non-transfected cells and SIF formation was quantified (100 cells analyzed for each of three biological replicates, statistical analysis was performed by one-way ANOVA versus WT and significances are indicated as follows: * = p<0.05, n.s. = not significant). SIF formation in HeLa cells transfected with pmCherry-ATG5-K130R is reduced. C–F) CLEM of HeLa cells transfected with pmCherry-ATG5-K130R. ATG5-K130R expressing cells showed no obvious changes in morphology as determined by light microscopy (C, D) and TEM (E, F). Note the double membrane structure for LAMP1-positive SIF (F). A cell representative of two biological replicates is shown (1–3 technical replicates with 2–3 cells each). Scale bars: 5 µm (A), 10 µm (B), 1 µm (D, E), 500 nm (F). (TIF) [file ppat.1004374.s009.tif]

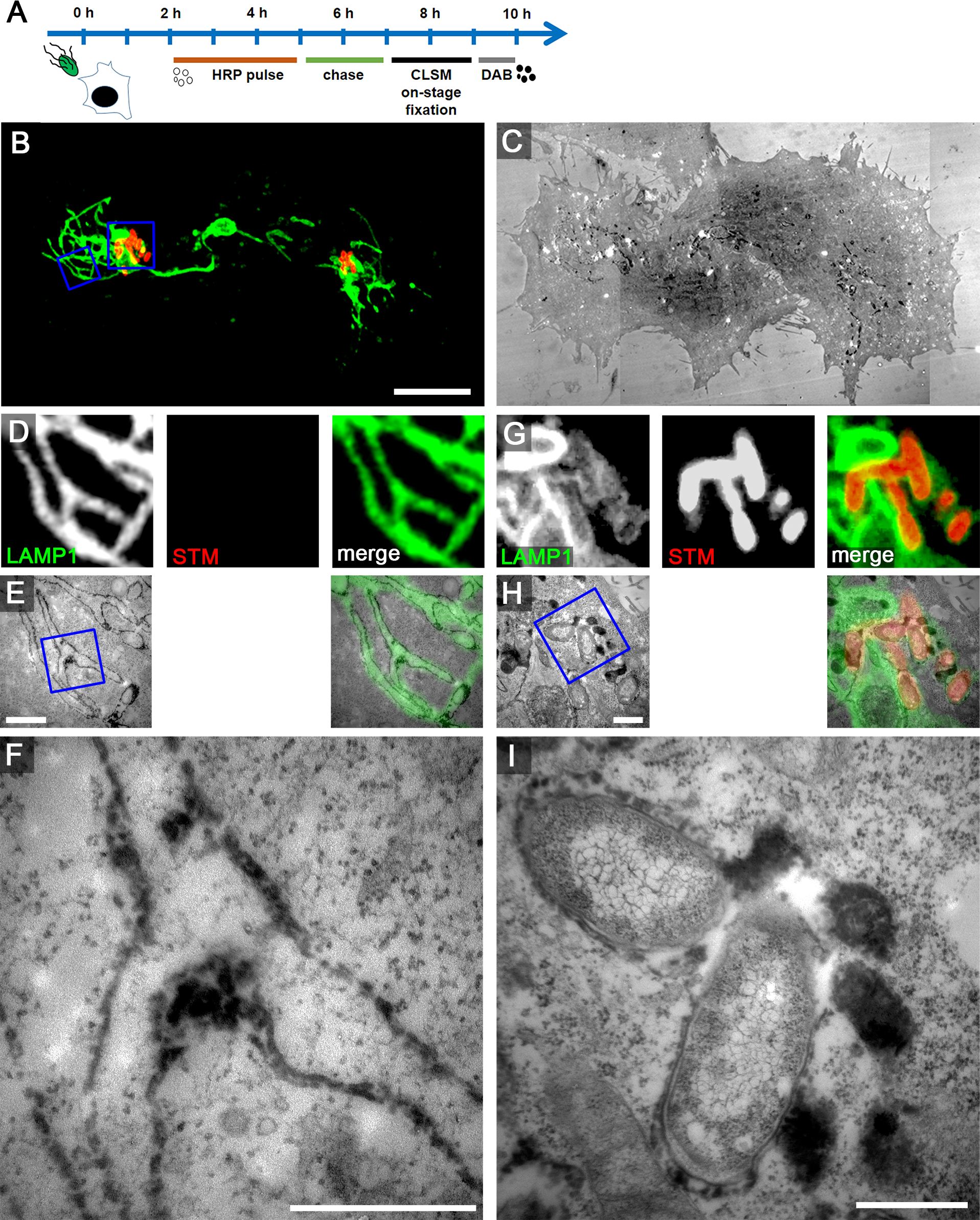

Supplement: Figure S10 — The outer lumen of double membrane SIF is accessible to endocytosed material. A) Scheme of the experiment. HeLa cells expressing LAMP1-GFP (green) were seeded on a petri dish with a gridded coverslip. Cells were infected with Salmonella WT expressing mCherry (STM, red). HRP was added as fluid tracer to the medium 2–5 h p.i. At 8 h p.i., selected cells were imaged by light microscopy (B, CLSM, MIP) and immediately fixed on stage. DAB conversion by HRP was performed and cells were prepared for TEM. Several images of the same section were stitched for an overview (C). Details of LAMP1-positive SIF are shown on fluorescence images (D, G, single Z plane), and TEM micrographs (E, F, H, I). I) Note DAB deposition between the two adjacent membranes of SIT (F) and within lumen of the SCV in direct contact with bacteria (I). A cell representative of four biological replicates is shown (1–3 technical replicates with 2–4 cells each). Scale bars: 10 µm (B, C), 1 µm (D, E, G, I, J, K), 500 nm (F, G). (TIF) [file ppat.1004374.s010.tif]

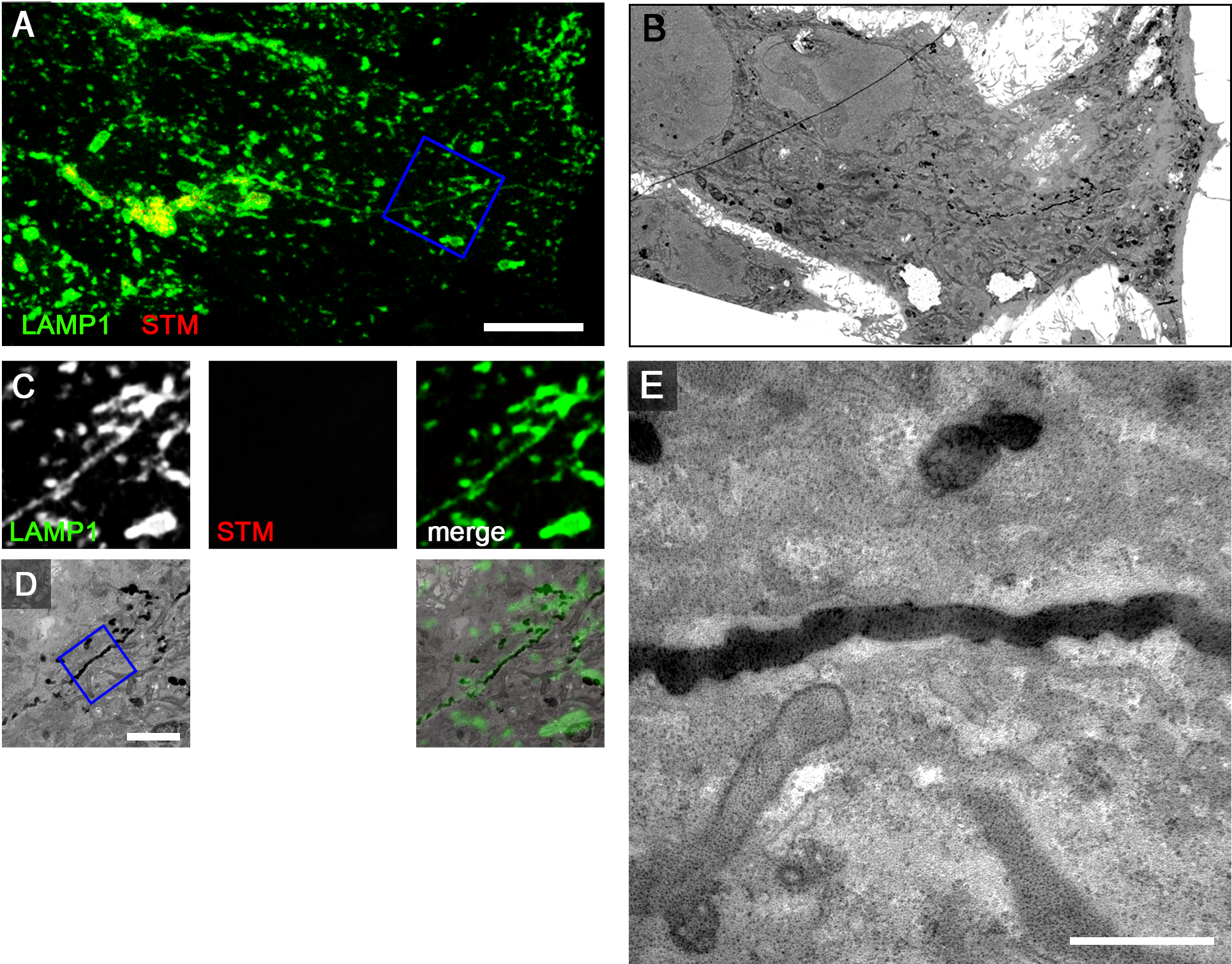

Supplement: Figure S11 — Early-stage SIF in HeLa cells are in interchange with endocytosed material. The experimental set-up was as described for Figure S10, but with HRP pulse/chase for 3 h and DAB conversion by HRP after on-stage fixation. Panel E) shows the presence of the DAB polymer within the whole lumen of single membrane SIF. A cell representative for two biological replicates is shown (1–2 technical replicates with 2–4 cells each). Scale bars: 10 µm (A, B), 2 µm (C, D), 500 nm (E). (TIF) [file ppat.1004374.s011.tif]

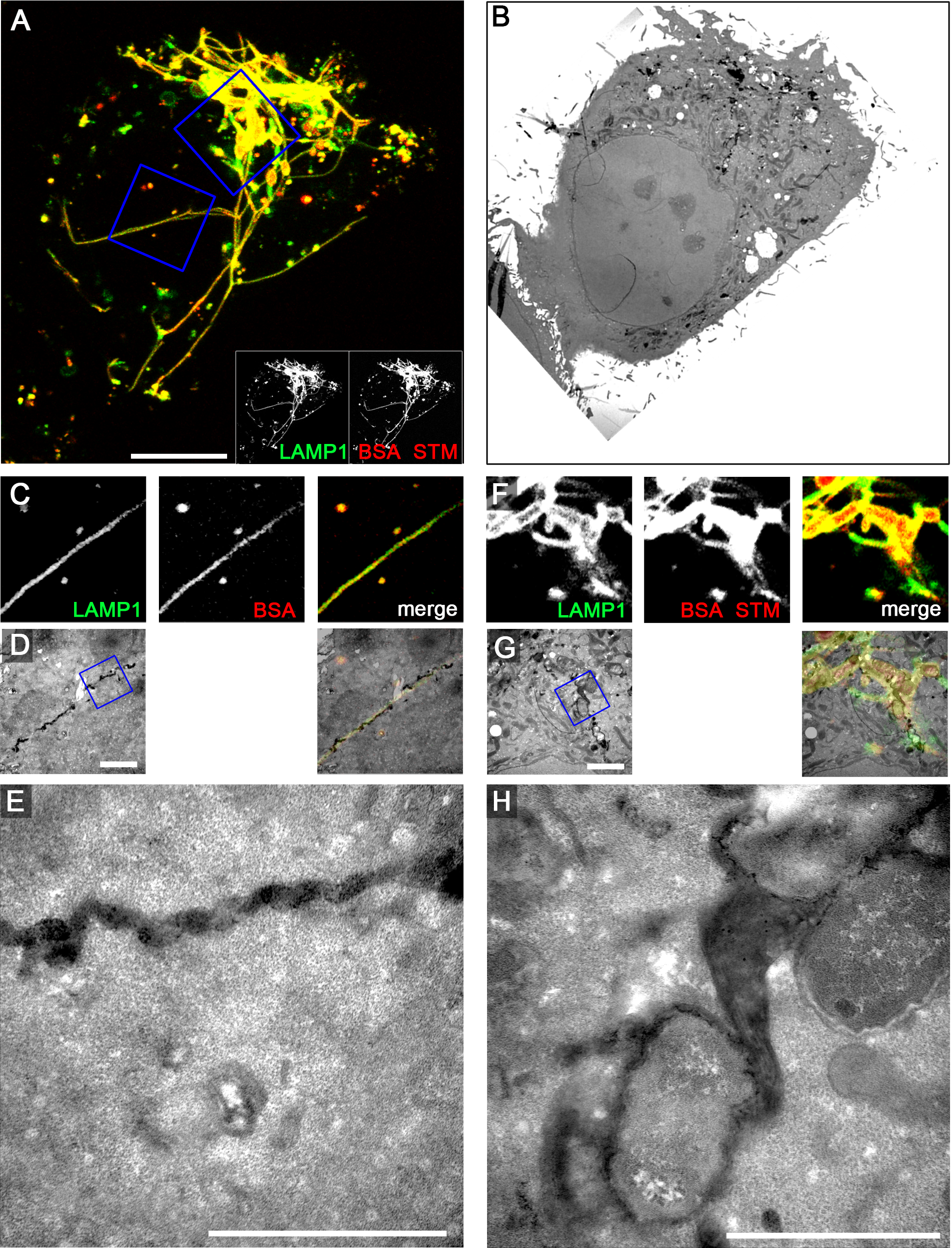

Supplement: Figure S12 — The SPI2-T3SS effector SseF is required for induction of double membrane SIT. HeLa cells expressing LAMP1-GFP (green) were seeded on a petri dish with a gridded coverslip. After infection with the Salmonella sseF-deficient strain expressing mCherry (STM, red), cells were pulse-chased with BSA-Rhodamine 2–5 h p.i. After live cell imaging at 8 h p.i. by CLSM (A, MIP), cells were fixed immediately on stage. Finally, DAB photo-conversion by Rhodamine was performed and samples were prepared for TEM. A) sseF-infected HeLa cells exhibit thin LAMP1-positive, BSA-Rhodamine-positive tubules. B) TEM micrograph of the same cell. CLEM of two different magnified ROIs showing only SIF (C, D, single Z plane) and Salmonella within SCV (F, G, single Z plane). E, H) Magnifications of structures of interest. Tubular structure with single membrane and DAB polymer inside the whole tubule lumen (E). Salmonella inside SCV with DAB polymer inside SCV (H). A cell representative for two biological replicates is shown (1–3 technical replicates with 2–3 cells each). Scale bars: 10 µm (A), 2 µm (D, G), 1 µm (E). (TIF) [file ppat.1004374.s012.tif]

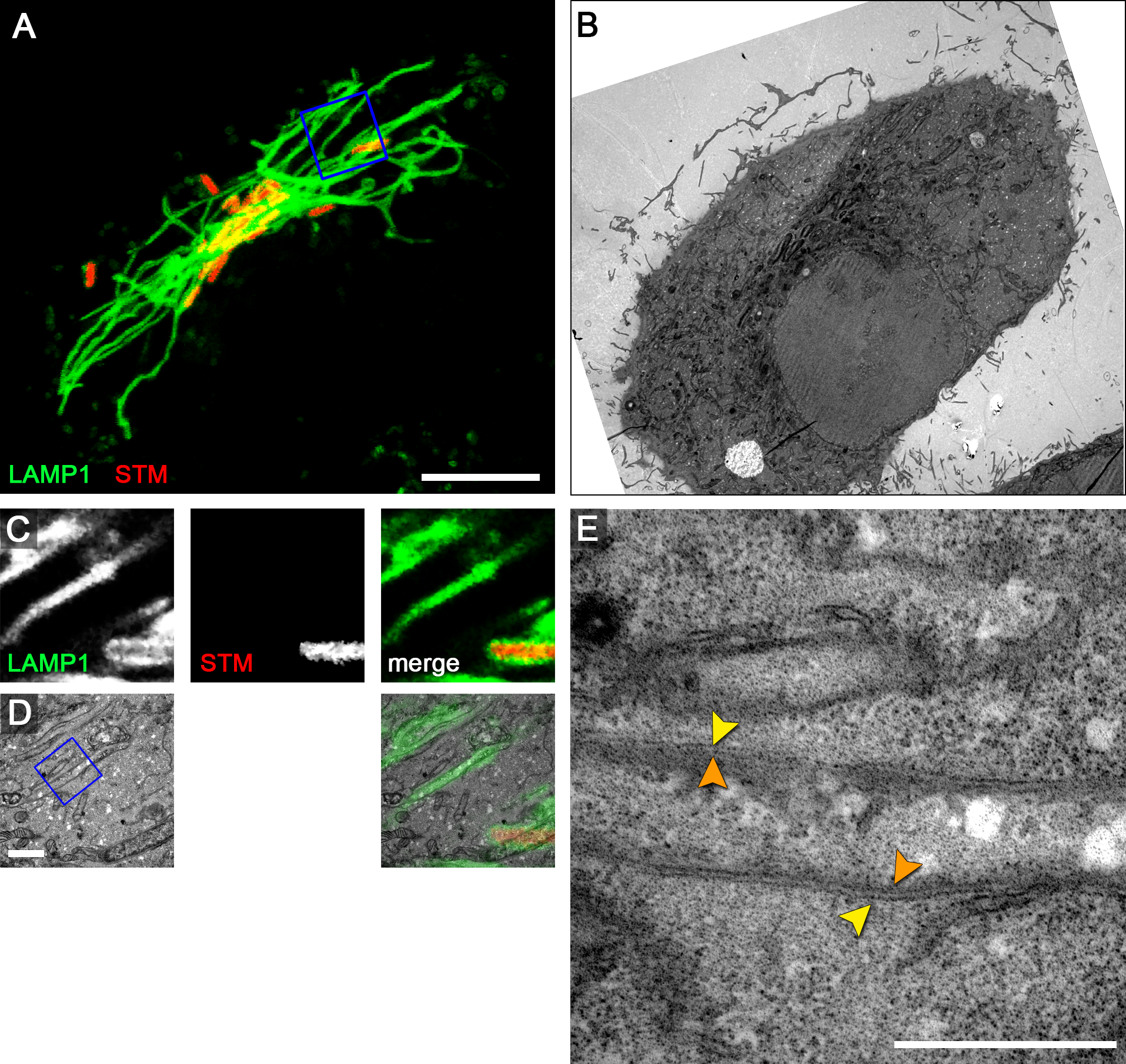

Supplement: Figure S13 — Complementation of the sseF strain with WT sseF restores induction of double membrane SIF. Infection and imaging was performed as for Figure 8, but the sseF mutant strain complemented with WT sseF expressing mCherry (STM, red) was used. The complementation of sseF leads to LAMP1-positive double membrane tubule in infected HeLa cells. Live cell imaging at 8 h p.i. (A, MIP of CLSM), low magnification TEM (B), details (C, single Z plane of CSLM; D, TEM) and higher magnification TEM (E) of a SIF. A cell representative for two biological replicates is shown (1–2 technical replicates with 2–4 cells each). Scale bars: 10 µm (A, B), 1 µm (C, D), 500 nm. (TIF) [file ppat.1004374.s013.tif]

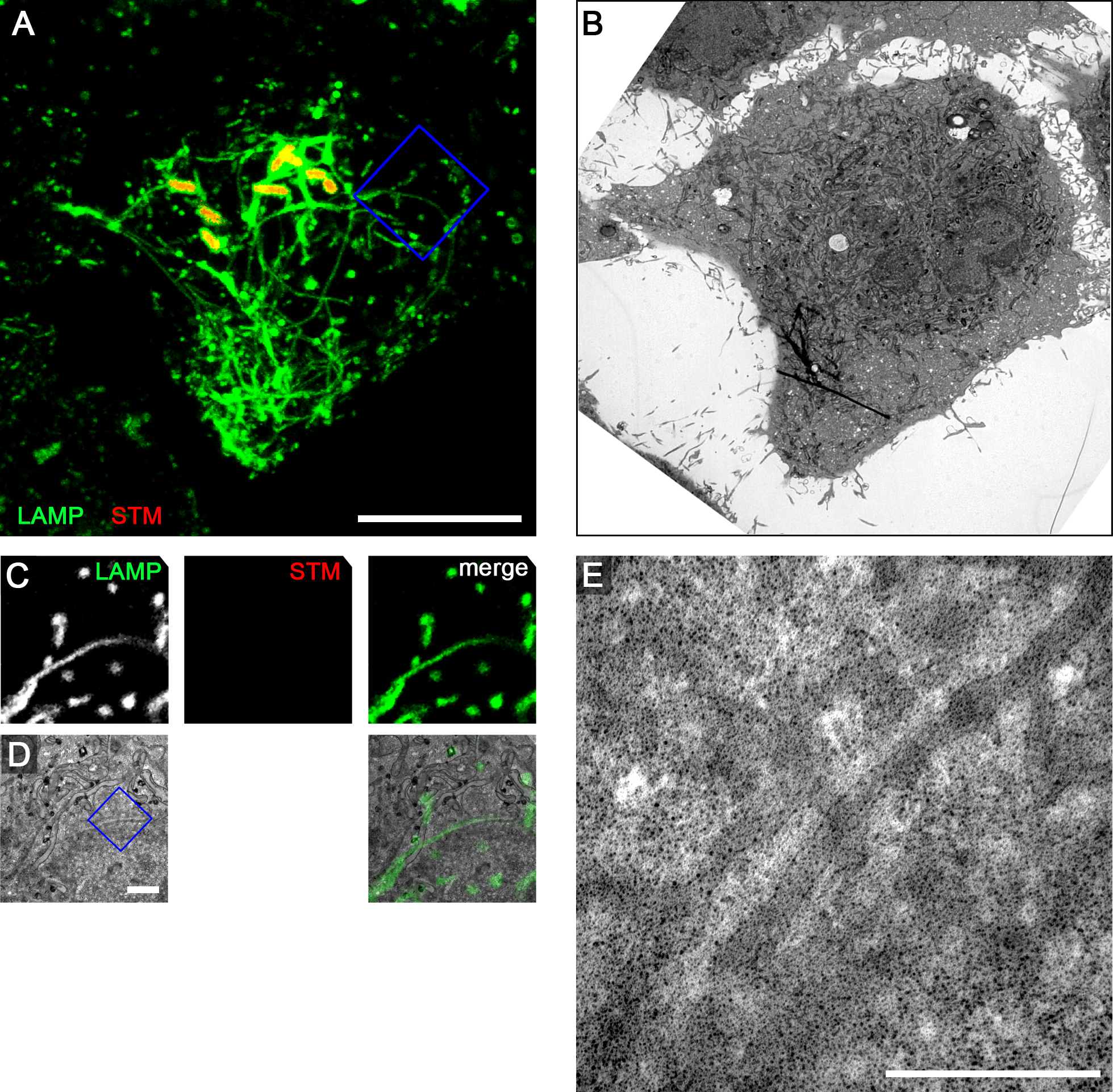

Supplement: Figure S14 — Complementation of the sseF strain with sseF Δ200–205 fails to restore double membrane SIF formation. Infection and imaging was performed as for Figure 8, but the sseF mutant strain complemented with sseF Δ200–205 expressing mCherry (STM, red) was used. The ΔsseF strain expressing sseF Δ200–205 leads to LAMP1-positive single membrane tubules (white arrowheads) in infected HeLa cells. Live cell imaging at 8 h p.i. (A, MIP of CLSM), low magnification TEM (B), details (C, single Z plane of CSLM; D, TEM) and higher magnification TEM (E) of a SIT. A cell representative of two biological replicates is shown (1–2 technical replicates with 2–4 cells each). Scale bars: 10 µm (A, B), 1 µm (C, D), 500 nm (E). (TIF) [file ppat.1004374.s014.tif]

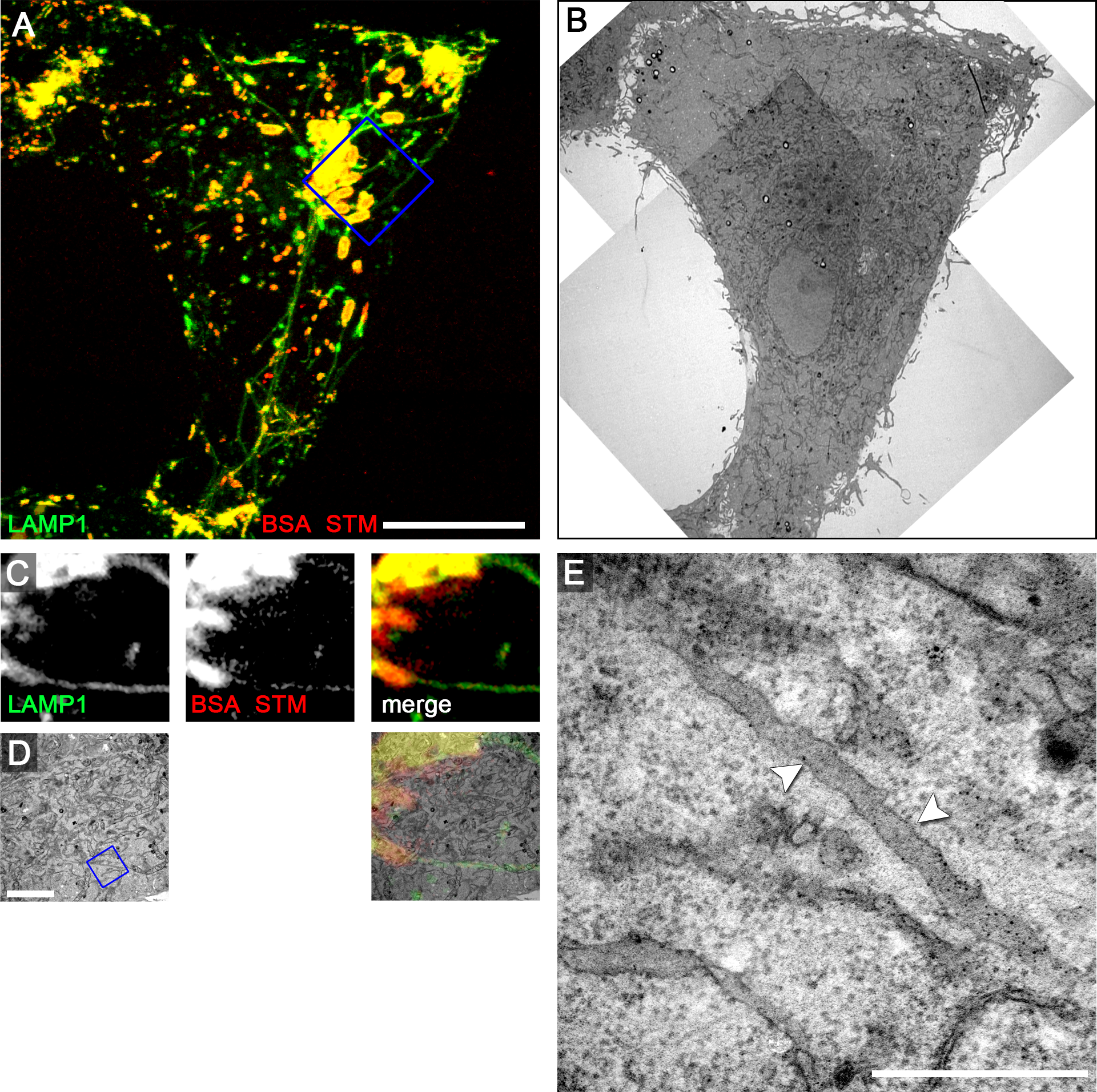

Supplement: Figure S15 — The SPI2-T3SS effector SseG is required for induction of double membrane SIF. Infection and imaging was performed as for Figure 8, but the sseG mutant strain expressing mCherry (STM, red) was used. HeLa cells infected with the ΔsseG strain exhibit thin LAMP1-positive tubules composed of a single membrane. Live cell imaging at 8 h p.i. (A, MIP of CLSM), low magnification TEM (B), details (C, single Z plane of CSLM; D, TEM) and higher magnification TEM (E) of a SIF. A cell representative of two biological replicates is shown (1–2 technical replicates with 2–3 cells each). Scale bars: 10 µm (A, B), 2 µm (C, D), 500 nm (E). (TIF) [file ppat.1004374.s015.tif]

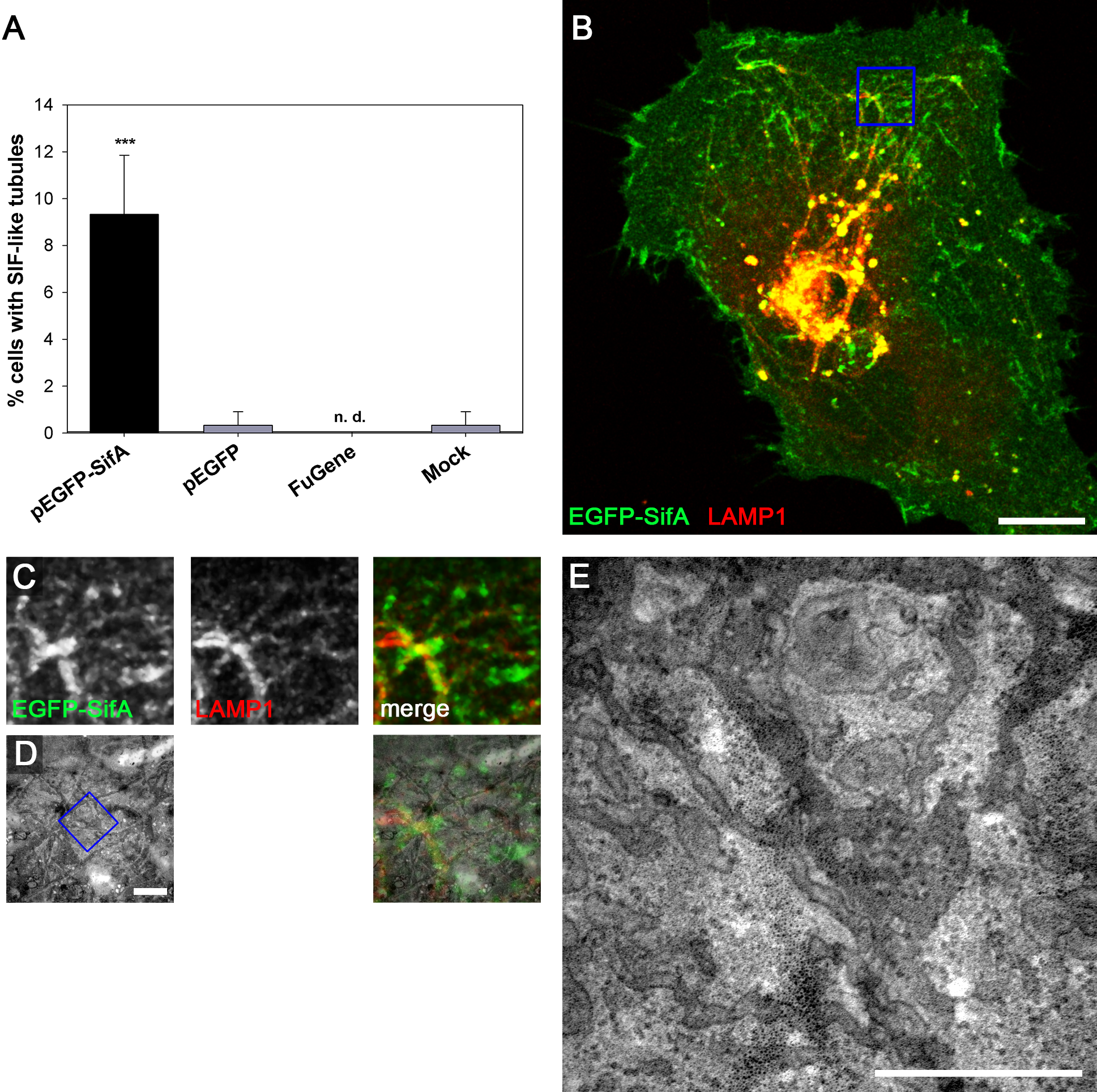

Supplement: Figure S16 — Expression of SPI2-T3SS effector sifA in HeLa cells leads to formation of SIF-like LAMP1-positive single membrane tubules. A) HeLa cells were transfected with pEGFP-SifA (green), the control vector pEGFP, transfection reagent FuGene only, or mock transfected. The next day, pulse-chase with Dextran-Alexa 568 for 3 h (red) was performed and for each condition at least 100 living cells were scored for presence of SIF-like tubules. Three biological replicates were performed, and statistical significances between pEGFP-SifA-transfected cells and other treatments were calculated by one-way ANOVA, and are indicated by *** = p<0.001, n.d. = not detectable. B–E) HeLa cells were cotransfected with pEGFP-SifA (green) and pLAMP1-mCherry (red) and subjected to CLEM the next day. After live cell imaging by CLSM (B, MIP) cells were immediately fixed on stage. HeLa cells showed many very thin tubular structures positive for EGFP-SifA and LAMP1-mCherry. Details of tubules are shown by correlative live cell CLSM (C, single Z plane) and TEM (D) micrographs. E) Higher magnifications of a network of tubules. Note the single membrane structure of the very thin tubules (arrowheads). A cell representative of three technical replicates with 2–4 cells each is shown. Scale bars: 10 µm (B), 1 µm (C, D), 500 nm (E). (TIF) [file ppat.1004374.s016.tif]
