# Supplementary material for: Reorganization of the Endosomal System in Salmonella-Infected Cells: The Ultrastructure of Salmonella-Induced Tubular Compartments
Source: PLoS Pathog. 2014 Sep 25;10(9):e1004374. doi: 10.1371/journal.ppat.1004374 (PMC4177991; doi:10.1371/journal.ppat.1004374)
Supplement: Text S1 — Stable cell line generation. (DOCX) [file ppat.1004374.s031.docx]

Text S1: **Stable cell line generation.** To generate a HeLa cell line stably expressing LAMP1‐GFP, the lentiviral vector system was used. For this purpose, the plasmid p3735 was cloned on the basis of L22_eGFP. L22_eGFP was cut with PacI and EcoRI. The insert CMV‐mcs‐Egfp was generated by PCR from pEGFP‐N1 with the primers CMV_for_PacI and EGFP_rev_MunIHpaI. The digestion of the PCR product with PacI and MunI was done using the CloneJet PCR Cloning Kit (Thermo Fisher Inc.) as an intermediate step. By ligation of the vector and the insert L22_cmvEgfp.N1 was generated. The following ligation of L22_cmvEgfp.N1 and pEgfpN1Hlamp, both cut before with EcoRI and BamHI, resulted in the plasmid p3735. About 2 x 10^6^ HEK 293FT cells were seeded two days before transfection in a cell culture-treated 10 cm diameter dish. The transfection of the cells was performed using the calcium phosphate method with 3.75 μg of L3 pCMV D8.2, 1.5 μg of L4 pHCMV‐G and 5 μg of p3735 in 500 μl transfection of each solution A and B. Three days after transfection, the virus particles were harvested by filtering the medium through a 0.45 μm filter and centrifugation at 77,000 x g for 1.5 h. The pellet was solubilized in 100 μl PBS and frozen in aliquots at ‐70°C. HeLa cells, seeded in a 6-well plate, were infected with 20 μl of the virus particles. After concentrating the infected cells via sorting in a FACSAria (BD) according to their green fluorescence (GFP), a mixed population with non‐fluorescent and fluorescent cells with different expression levels was obtained. To obtain populations of cells with low, middle, high and very high GFP expression levels it was necessary to increase the number of cells before re-sorting. The second round of FACS sorting was performed using a PE laser. The cells were sorted into five different fractions: P1, the fraction of non‐fluorescent cells (waste), P2, P5, P6 and P7 as fractions containing cells with increasing LAMP1‐GFP expression levels. Before proceeding further, the heterogeneous fractions were tested for the ability of the LAMP1‐GFP-expressing cells to form SIFs upon *Salmonella* infection. The mixed populations were then diluted and plated in 6‐well plates. Following growth of individual cells to colonies, individual clones were picked with the help of a pipette under the microscope. The clones were grown in 24‐well plates to increase cell numbers by further culturing. Following tests for the SIF formation by both live cell imaging and immunostaining with antibodies against human LAMP1, four clones (P6‐9, P6‐10, P7‐11, and P7‐20) were finally selected.
